# Assessing the inhibition efficacy of clinical drugs against the main proteases of SARS‐CoV‐2 variants and other coronaviruses

**DOI:** 10.1002/qub2.60

**Published:** 2024-07-06

**Authors:** Wenlong Zhao, Cecylia S. Lupala, Shifeng Hou, Shuxin Yang, Ziqi Yan, Shujie Liao, Xuefei Li, Nan Li

**Affiliations:** ^1^ Key Laboratory of Quantitative Synthetic Biology Shenzhen Institute of Synthetic Biology Shenzhen Institutes of Advanced Technology Chinese Academy of Sciences Shenzhen China; ^2^ University of Chinese Academy of Sciences Beijing China

**Keywords:** drug resistance, enzymatic activity, main protease, SARS‐CoV‐2

The rapid evolution of severe acute respiratory syndrome coronavirus 2 (SARS‐CoV‐2) mainly due to its high mutation rate and rapid viral replication, has led to new variants resistant to the available vaccines and monoclonal antibodies. In contrast, oral clinical drugs targeting viral protease and RNA polymerase remain effective against Omicron variants [1]. Main protease (M^pro^) plays a crucial role in the maturation and replication of viral strains, making it an attractive target for developing antiviral drugs. Nirmatrelvir (NTV) is the first‐in‐class M^pro^ peptidomimetic covalent inhibitor known as “Paxlovid” approved in 2021 by the Food and Drug Administration [2]. Nevertheless, NTV‐resistant M^pro^ mutants particularly the E166V mutation, have been characterized in the Global Initiative on Sharing Avian Influenza Data (GISAID) database [3] and reported in COVID‐19 patients [4, 5]. Additionally, viral passage experiments have identified other mutations such as L50F and T21I, which can restore the viral fitness reduced by E166V [6]. The second‐generation M^pro^ drug, ensitrelvir (ETV), is a non‐covalent inhibitor approved in 2022 with the brand name “Xocova” [7]. Besides, leritrelvir (LTV) is another covalent inhibitor that was approved in China last year [8]. Preclinical studies showed that ETV and LTV exhibited comparable antiviral activity as NTV and improved pharmacokinetics. However, the effectiveness of these clinical drugs against NTV‐resistant M^pro^ mutants has yet to be fully assessed. Here, we analyzed the inhibition efficiency of four inhibitors, NTV, ETV, LTV, and a veterinary drug, GC376 (Figure 1A), against the M^pro^ of SARS‐CoV‐2 variants and other pathogenic coronaviruses.

**FIGURE 1:**
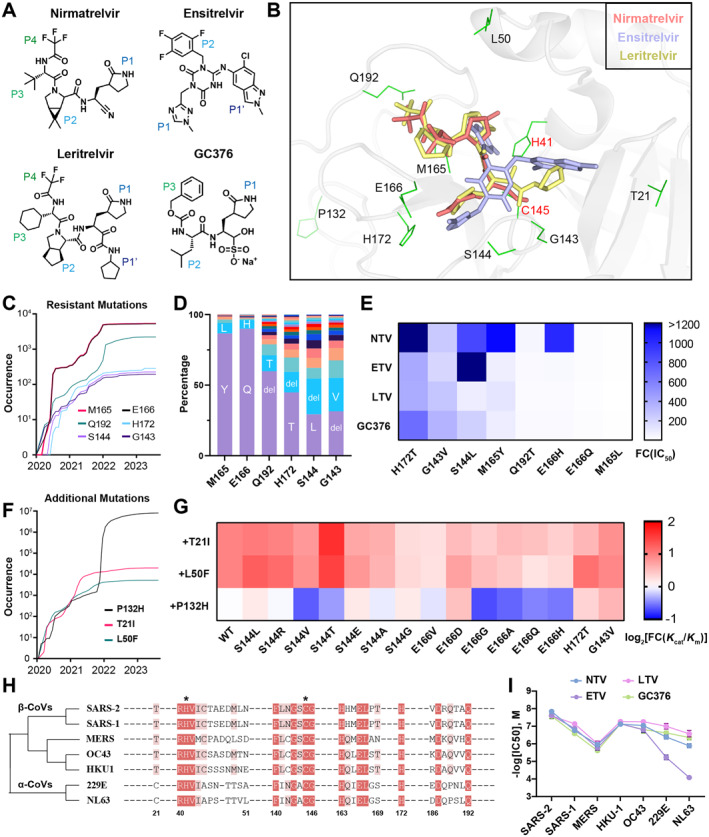
Assessment of drug inhibition against M^pro^ of SARS‐CoV‐2 mutants and other coronaviruses. (A) Chemical structure of four M^pro^ inhibitors. Their pocket‐binding moieties including P1′ and P1–4 are labeled in gradient colors. (B) Binding mode of nirmatrelvir (PDB code: 7VH8), ensitrelvir (PDB code: 7VU6), and leritrelvir (PDB code: 8IGN) in the SARS‐CoV‐2 M^pro^ pocket. The surrounding residues analyzed in this study are labeled and two active sites, H41 and C145, are colored in red. (C) Longitudinal analysis of the accumulation of global mutations at six residues of M^pro^ from January 2020 to September 2023, as per the GISAID database. Note the overlap of two curves, E166 and M165. (D) Distribution of different mutation types at six residues, with frequent mutation types labeled in the center. (E) Fold‐change in IC_50_ relative to wild‐type M^pro^ for the indicated mutants, determined through a FRET‐based enzymatic assay with three replicates. (F) Longitudinal analysis of three M^pro^ mutations using the same method as in (C). (G) Fold‐change (log base 2) in *K*_cat_/*K*_m_ of double mutations that consist of additional mutations, T21I, L50F, and P132H, relative to single mutations, as determined by a FRET‐based enzymatic assay with three replicates. (H) Phylogenetic relationship and protein sequence alignment of seven β‐ and α‐coronaviral M^pro^. The conserved amino acids are colored with gradual degrees of red and two active sites are designated with asterisks above. (I) IC_50_ of four M^pro^ inhibitors against various coronaviral proteases. The data represent mean ± SEM from three experiments. FRET, fluorescence resonance energy transfer; GISAID, Global Initiative on Sharing Avian Influenza Data.

The M^pro^ drugs interact tightly with the amino acids of the active pocket (Figure 1B), and nonsynonymous mutations of pocket residues have the potential to induce severe resistance than mutations in other locations [3]. Particularly, six pocket residues, G143, S144, M165, E166, H172, and Q192S have been reported to confer SARS‐CoV‐2 resistance to NTV [3]. Based on the GISAID database, we investigated the occurrence and frequency of mutations at these six residues. Results showed that all of these sites have been consistently mutating since 2020 (Figure 1C) and adjacent residues, such as M165/E166 and G143/S144, exhibited similar mutational rates. The substitutions at these residues demonstrated a preference for specific types of amino acid, for example, the substitution of glutamine (Q) or histidine (H) at position E166 (Figure 1D). These mutations are thought to confer resistance to SARS‐CoV‐2 and simultaneously compromise viral fitness [3].

To assess the impact of substitutions at the six residues on drug inhibition, we purified 30 tag‐free recombinant M^pro^ mutants that exhibited high frequencies at these mutation sites (Figure S1A). Fluorescence resonance energy transfer (FRET) assays were then conducted to determine their IC_50_, *K*_i,_ and *K*_cat_/*K*_m_ (Figure S2 and Table S1). The results showed that the IC_50_ of NTV increased remarkably in H172T, S144L, M165Y, and E166H mutants (Figure 1E). Their IC_50_ values exceeded 10 μM (>500‐fold of wild‐type M^pro^), indicating a severe resistance to NTV. By contrast, these mutants remained susceptible to ETV (except S144L), LTV, and GC376. Further analysis revealed that mutations at E166 and S144 significantly confer resistance to NTV and ETV, respectively (Figure S3). The resistance of E166D/V/H substitutions to NTV is consistent with previous studies and could be attributed to the hydrogen bond between E166 and the lactam nitrogen of NTV at the P1 position [2]. Unlike NTV, the central triazine moiety of ETV forms a distinctive hydrogen bond with the backbone nitrogen of E166, thereby preserving the binding affinity of ETV regardless of substitutions at E166 (Figure S4A). Nevertheless, hydrophobic S144/V/L substitutions significantly impeded the efficacy of ETV, resulting from the diminished interaction between its triazine moiety and the hydroxyl group of S144 at the S1 pocket [7].

By contrast, LTV and GC376 remained effective to S144 and E166 mutants. Although they share a common lactam ring at the P1 position as NTV, they differ especially in their warheads. LTV and GC376 possess ketoamide and aldehyde warheads, respectively, as opposed to NTV, which utilizes a nitrile warhead. Previously resolved co‐structure of M^pro^ with LTV [8] showed that the α‐ketoamide warhead of LTV can interact with the S1′ pocket, forming a hydrogen bond with H41 and hydrophobic contact with L27. Our molecular docking indicated that this warhead at the P1′ position potentially contributed to greater binding affinity than the pharmacophore at the P1 position (Figure S4). Additionally, GC376 remained effective to various types of M^pro^ mutants in our study. The potency of GC376 probably benefits from its bisulfite warhead which is more reactive than that of NTV and LTV.

To validate the resistance of these mutants in cellular models, we further assessed the inhibition efficiency of M^pro^ inhibitors using the flip green fluorescent protein (FlipGFP) assay [9]. The results showed that the GFP/mCherry fluorescence ratio associated with the activity of E166V/H mutants cannot be attenuated by NTV, which means that these mutants are resistant to NTV, instead of ETV and LTV (Figure S5). Nevertheless, it is difficult for the FlipGFP model to analyze M^pro^ mutants with lower enzymatic activity, such as S144L, because they were not able to produce detectable fluorescence signals. Future studies should consider using alternative cellular models such as those utilizing luciferase or recombinant viruses. Overall, four M^pro^ inhibitors, NTV, ETV, LTV, and GC376, showed different resistance profiles. Importantly, LTV and GC376 remained effective for some NTV‐ and ETV‐resistant mutants.

In addition to structural molecular interactions between M^pro^ and inhibitors, pharmacokinetics also plays a crucial role in determining antiviral activity. NTV, a representative peptidomimetic covalent inhibitor, requires co‐administration with ritonavir, a CYP3A4 inhibitor, to enhance its in vivo half‐life [2]. In contrast, ETV, a second‐generation M^pro^ inhibitor, the only non‐covalent drug in our study, exhibits more favorable pharmacokinetic profiles and no longer necessitates ritonavir co‐administration [7]. Furthermore, α‐ketoamide inhibitors, such as LTV, demonstrate improved pharmacokinetics [8], with a longer half‐life (4.8 h) than NTV (0.5 h) and ETV (2.4 h) [7] in male rat models following intravenous administration. Previous research studies indicate that LTV exhibits “slow‐on, slow‐off” kinetic behavior, forming a stable enzyme‐inhibitor complex. This characteristic prolongs the drug‐target residence time of LTV, potentially enhancing its inhibitory activity against M^pro^ mutants [8]. Given the distinct pharmacokinetic profiles of these M^pro^ inhibitors, further studies are warranted to evaluate their antiviral efficacy against SARS‐CoV‐2 with resistance mutations in animal models.

The replication of SARS‐CoV‐2 depends on the enzymatic activity of M^pro^. Our FRET experiments show that the wild‐type M^pro^ has a higher K_cat_/K_m_ value than other resistant mutants (Figure S6), which means that resistant mutations could attenuate the catalytic activity. Particularly, we observed that H172T and G143V mutants exhibited severe resistance to all drugs (Figure 1E), but their enzymatic activities significantly declined (*K*_cat_/*K*_m_ < 10). This result implies that the resistance level of M^pro^ mutants is inversely correlated with the enzymatic activity to some degree. Previous research studies found that impaired activity of resistant M^pro^ can be compensated by secondary mutations, such as T21I and L50F, increasing the viral replication efficiency [3]. Furthermore, P132H is the most prevalent substitution mutation in M^pro^, and demonstrates comparable enzymatic activity as the wild‐type M^pro^ [10]. But it remains unclear whether it would synergize with other resistant mutants to affect enzymatic activity.

To evaluate the effect of three additional mutations, we prepared 45 purified M^pro^ with double mutations, composed of T21, L50F, and P132H, respectively (Figure 1F and Figure S1B). By comparing the enzymatic activities of resistant mutants with or without three additional mutations, we found that T21I and L50F, instead of P132H, were able to enhance the enzymatic activity of all mutants studied (Figure 1G). Moreover, the introduction of T21, L50F, and P132H had little impact on drug resistance (Figure S7). These results highlight the compensatory function of secondary mutations for the activity of resistant M^pro^.

Apart from SARS‐CoV‐2, the main protease is also an attractive target for other pathogenic α‐ and β‐coronaviruses (Figure 1H). To assess the anti‐coronaviral activity of NTV, ETV, LTV, and GC376, we purified six coronaviral M^pro^ and conducted the FRET assay to compare their drug inhibition. The results showed that four inhibitors exhibited comparable efficacy against the β‐coronaviral M^pro^, but LTV showed better broad‐spectrum activity, especially for α‐coronaviruses, 229E and NL63 (Figure 1I and Figure S8). The effectiveness of LTV probably benefits from its α‐ketoamide warhead that forms two hydrogen bonds with conserved active residues, His and Cys, which are necessary for the activity of coronaviral M^pro^ (Figure S9B).

However, ETV showed less efficacy for α‐coronavirus (Figure 1I), which was consistent with reported cytopathic effect assays, showing that the EC_50_ value of ETV against 229E is higher than other β‐coronaviruses [7]. The reduced binding affinity of ETV could be attributed to the difference between α‐ and β‐coronaviral M^pro^ pockets. Though the substrate‐binding residues of M^pro^ are highly conserved among different coronaviruses, the α‐coronaviral proteases possess a more negatively charged and open pocket than β‐coronaviruses, including SARS‐CoV‐2 and Middle East respiratory syndrome coronavirus (MERS‐CoV) (Figure S9A). In addition, α‐coronaviruses carry different residues from SARS‐CoV‐2 at S144 and M49, which play a vital role in ETV–pocket interaction (Figure S9C). Interestingly, ETV was also more sensitive to SARS‐CoV‐2 mutants at residue S144 and M49 than other drugs, as revealed by our study and previous research studies [11]. These results implied that the resistance profiles of M^pro^ inhibitors could reflect their inhibitor–pocket interactions.

Taken together, our study characterized the resistance profiles of four M^pro^ inhibitors. LTV, a newly approved drug, and GC376 remained effective for the M^pro^ mutants that were resistant to NTV and ETV. Although resistant mutations compromised the enzymatic activity, additional mutations, T21I and L50F, were able to broadly compensate the M^pro^ activity associated with viral replication efficiency. Moreover, LTV showed better broad‐spectrum activity for other pathogenic coronaviruses, probably due to its α‐ketoamide warhead interacting with conserved residues. Overall, this study offered valuable insights for the development of next‐generation M^pro^ inhibitors for SARS‐CoV‐2 variants as well as other coronavirus diseases and highlighted the importance of monitoring resistant variants harboring compensatory mutations.

## AUTHOR CONTRIBUTIONS

**Wenlong Zhao**: Conceptualization; methodology; writing – original draft. **Cecylia S. Lupala**: Methodology; writing – review & editing. **Shifeng Hou**: Investigation; validation. **Shuxin Yang**: Methodology; validation. **Ziqi Yan**: Validation. **Shujie Liao**: Validation. **Xuefei Li**: Project administration; supervision; writing – review & editing. **Nan Li**: Project administration; supervision; writing – review & editing.

## CONFLICT OF INTEREST STATEMENT

The authors declare no competing interests.

## Supporting information

Supporting Information S1

## Data Availability

All data in this study are available within the article or from the corresponding author upon reasonable request.
