# Supplementary material for: Assessing the inhibition efficacy of clinical drugs against the main proteases of SARS‐CoV‐2 variants and other coronaviruses
Source: Quant Biol. 2024 Jul 6;12(3):324–8. doi: 10.1002/qub2.60 (PMC12806454; doi:10.1002/qub2.60)
Supplement: Supplementary file 1 — Supporting Information S1 [file QUB2-12-324-s001.docx]

**Supplementary Materials for**

**Assessing the inhibition efficacy of clinical drugs against the main proteases of SARS-CoV-2 variants and** **other coronaviruses**

Wenlong Zhao^1,2^, Cecylia S. Lupala^1^, Shifeng Hou^1^, Shuxin Yang^1^, Ziqi Yan^1^, Shujie Liao^1,2^, Xuefei Li^1*^ and Nan Li^1*^

1. *Key Laboratory of Quantitative Synthetic Biology, Shenzhen Institute of Synthetic Biology, Shenzhen Institutes of Advanced Technology, Chinese Academy of Sciences, Shenzhen 518055, China*
2. *University of Chinese Academy of Sciences, Beijing 101408, China*

**^*^**Correspondence: nan.li@siat.ac.cn; xuefei.li@siat.ac.cn

**This PDF file includes:**

Materials and Methods

Fig. S1 to S9

Tables S1

**Materials and Methods**

M^pro^ mutation analysis

The nirmatrelvir-resistant SARS-CoV-2 M^pro^ mutations at six residues, G143, S144, M165, E166, H172, and Q192 were selected according to previous research^1,2^. The viral genome sequences of these M^pro^ mutants from January 2020 to September 2023 were downloaded based on the annotation of amino acid substitutions in the GISAID database (<https://gisaid.org>). They were filtered with “complete sequence” and “collection date complete”. The accumulated occurrence of M^pro^ mutation over the past 3 years was counted based on the collection date and was plotted as line charts by Prism 9 software. The proportion of different types of substitution or deletion at six mutation sites was calculated and plotted as a histogram by Prism 9 software.

Protein cloning, expression, and purification

Cloning and expression were performed as described in our previous research^3^. Full-length wild-type SARS-CoV-2 main protease (M^pro^) was cloned into the pET-28b vector, with N-terminal six-histidine (His) and small ubiquitin-like modifier (SUMO) tags. The recombinant M^pro^ was expressed in *E. coli* BL21(DE3) cells (Transgene). Transformed clones were grown in 250 ml LB medium with kanamycin (50 μg/ml) at 37℃. When the OD600 reached 0.6–0.8, the expression of the His-SUMO-tagged M^pro^ was induced by the addition of isopropyl β-D-1-thiogalactopyranoside (IPTG) to a final concentration of 0.2 mM. The BL21(DE3) cells were incubated at 16℃ for 18h. Bacterial cultures were harvested by centrifugation, and the pellet was resuspended in lysis buffer (20 mM Tris, 300 mM NaCl, 1 mM DTT, pH 8.0) and then lysed by high-pressure homogenization. The lysate was clarified by centrifugation at 15,000 g for 30 min, and the supernatant was incubated with 1ml Ni-NTA resin on a rotator at 4℃ for 1h. After the resin was thoroughly washed with 20mM imidazole in lysis buffer, His-tagged SUMO protease (Ulp1, purified by our laboratory) was added to a final concentration of 50 μg/ml in 3 ml lysis buffer to remove the His-SUMO tag at 4℃ overnight. Following the digestion, the His-tagged Ulp1 and His-SUMO tag still stayed in Ni-NTA resin, and the tag-free M^pro^ in the liquid phase was collected in lysis buffer. The purified recombinant proteins were fast frozen in liquid nitrogen and stored at −80℃.

For SARS-CoV-2 M^pro^ mutants, all single and double mutants were generated by site-directed mutagenesis based on the pET-28b plasmid expressing wild-type M^pro^ and verified by Sanger sequencing. For other coronaviral proteases, six full-length genes were optimized and synthesized for *E. coli* expression (Genscript), including the M^pro^ of SARS-CoV-1, MERS, OC43, HKU1, 229E, and NL63. The expression and purification steps were the same as wild-type M^pro^.

Enzymatic activity assay

For measurement of kinetics parameters (K_m_, V_max_, and K_cat_/K_m_) of SARS-CoV-2 M^pro^, the fluorescence resonance energy transfer (FRET) based substrate Dabcyl-KTSAVLQ↓SGFRKME-Edans (Beyotime, # P9733) was used in 50 μl assay buffer (20 mM Tris, 120 mM NaCl, 1 mM EDTA, 1 mM DTT, pH 6.5). Proteolytic reactions were conducted with optimized concentrations of M^pro^ proteins and series concentrations of substrate from 200 μM to 1 μM. Fluorescence readings were collected with filters for excitation at 360 nm and emission at 460 nm in a plate reader (BioTek Synergy4) after the addition of substrate. The initial velocity derived from the first 10 min of the kinetic progress curves was plotted against the substrate concentrations using the classic Michaelis−Menten equation in Prism 9 software.

Drug inhibition assay

Four M^pro^ inhibitors included in this study: nirmatrelvir (PF-07321332, #HY-138687) and ensitrelvir (S-217622, #HY-143216) were purchased from MedChemExpress. GC376 (#S0475) was purchased from Selleck. Leritrelvir was synthesized by Depu Biosciences (Shanghai).

For measurements of IC_50_ and K_i_, optimized concentrations of the M^pro^ proteins were incubated with a series concentration of inhibitors in 50 μl assay buffer for 5 min. The reaction was initiated by adding 20 μM FRET substrate and fluorescence signals were collected at 30℃ for 30 min to measure the initial velocity. The percentage of inhibition or activity was calculated based on control wells without inhibitor (0% inhibition/100% activity) or without enzyme (100% inhibition/0% activity). The IC50 values were calculated using a dose-response curve in Prism 9 software (log(inhibitor) vs. normalized response -- Variable slope). The K_i_ values were calculated using the Morrison equation (tight binding) with measured enzyme kinetics parameters in Prism 9 software. All values (K_cat_/K_m_, IC_50_, and K_i_) were determined in three replicates and summarized in Supplementary Table S1.

FlipGFP assay

The FlipGFP system included FlipGFP-T2A-mCherry and M^pro^ genes as described in previous research^4^. The SARS-CoV-2 M^pro^ cleavage site (AVLQ↓SGFR) was introduced into the FlipGFP protein, which makes active M^pro^ selectively recognize and cleave the FlipGFP to generate fluorescence signal. In addition, the mCherry signal was used as an internal control to normalize the FlipGFP signal, so the GFP/mCherry fluorescence ratio reflected the cellular activity of M^pro^. The full-length FlipGFP-T2A-mCherry gene was synthesized in pcDNA3.1 vector (Genscript). The wild-type M^pro^ gene was obtained from pET-28b-M^pro^ plasmid and introduced into the pcDNA3.1 vector by Gibson assembly and then M^pro^ mutants were generated by site-directed mutagenesis.

293T cells were seeded in 96-well plates overnight to reach 70-90% confluency. 50 ng FlipGFP plasmid and 50 ng M^pro^ plasmid were transfected into each well with Lipo293^TM^ transfection reagent (Beyotime, # C0521) according to the manufacturer’s protocol. 3 hours after transfection, 1 μl DMSO or testing inhibitors was directly added to wells. 48 hours after transfection, cell images were taken by Ti2-A fluorescence microscope (Nikon) via 10 × objective lens using GFP and mCherry channels. The mean fluorescence intensity of 293T cells was detected by Cytoflex-s flow cytometry (Beckman) using FITC and ECD channels and was analyzed by CytExpert software to calculate the GFP/mCherry fluorescence ratio.

Molecular docking and MMGBSA calculations

The crystal structures of M^pro^ in complex with the inhibitors Nirmatrelvir (PDB ID: 7EV8), Ensitrelvir (PDB ID: 7VU6), and Leritrelvir (PDB ID: 8IGN), were downloaded from the Protein Data Bank. Except for the ligands, water, and other co-crystallized molecules were removed. The proteins were prepared using the Protein Preparation Wizard in Maestro software (<https://schrodinger.com>) to ensure structural correctness for hydrogen consistency, bond orders, steric clashes, and charges. Protonation states of protein residues were calculated at a pH of 7.0. The covalent docking algorithm (CovDock) implemented in Maestro was then used to predict the binding poses. CovDock mimics covalent ligand binding by first positioning the pre-reaction form of the ligand in the binding site close to the receptor reactive residue with positional constraints and then generating the covalent attachment.

The binding pose of the covalently bound ligands was assessed using their scores (MMGBSA dG_Bind) and by comparing them to the poses in their native crystal structures. The molecular mechanics generalized Born surface area” (MMGBSA), implemented in the Prime module (Schrödinger) uses the OPLS-AA force field and VSGB 2.0 implicit solvation model to estimate the binding energy of the receptor-ligand complex. The binding energy is calculated as: $\Delta G \left( \mathrm{bind} \right)= E_{Complex}- \left( E_{Ligand} + E_{Receptor} \right)$.

All structural figures resulted were generated using Pymol software (<http://www.pymol.org>).

**References**

1. Hu, Y. *et al.* Naturally Occurring Mutations of SARS-CoV-2 Main Protease Confer Drug Resistance to Nirmatrelvir. *Acs Central Sci* **9**, 1658–1669 (2023).

2. Tzou, P. L. *et al.* Sierra SARS-CoV-2 sequence and antiviral resistance analysis program. *J Clin Virol* **157**, 105323 (2022).

3. Hu, F. *et al.* A novel framework integrating AI model and enzymological experiments promotes identification of SARS-CoV-2 3CL protease inhibitors and activity-based probe. *Brief Bioinform* **22**, bbab301 (2021).

4. Xia, Z. *et al.* Rational Design of Hybrid SARS-CoV-2 Main Protease Inhibitors Guided by the Superimposed Cocrystal Structures with the Peptidomimetic Inhibitors GC-376, Telaprevir, and Boceprevir. *ACS Pharmacol Transl Sci* **4**, 1408–1421 (2021).

**Fig. S1**


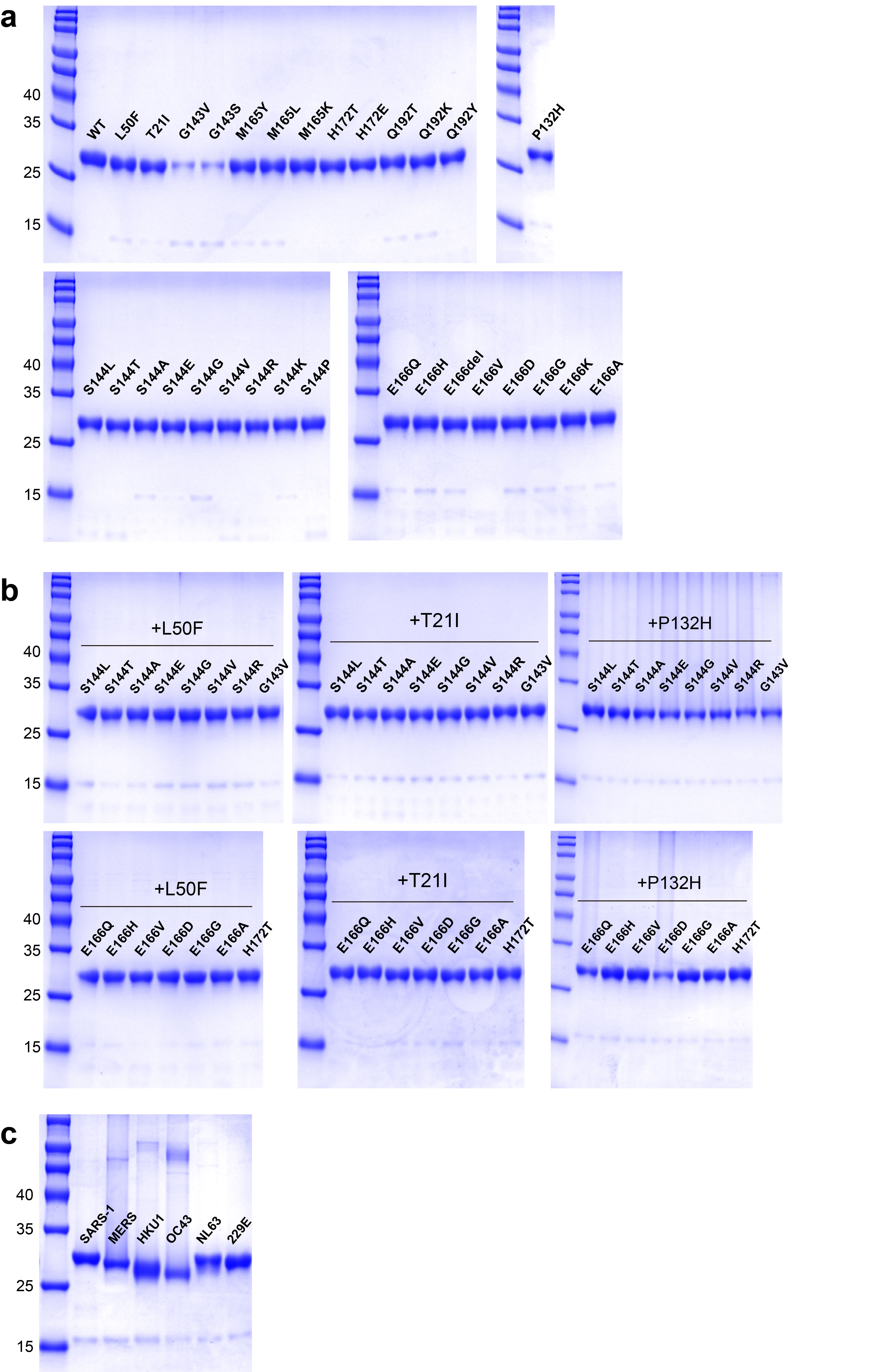


**Fig. S1 Purified tag-free recombinant M^pro^ proteins.** SARS-CoV-2 wild-type M^pro^ and single mutants (a), double mutants (b), and other coronaviral M^pro^ proteins (c) were expressed and purified in this study. 2 μg of recombinant proteins were analyzed on 12% sodium dodecyl sulfate-polyacrylamide gel by staining with Coomassie blue.

**Fig. S2**


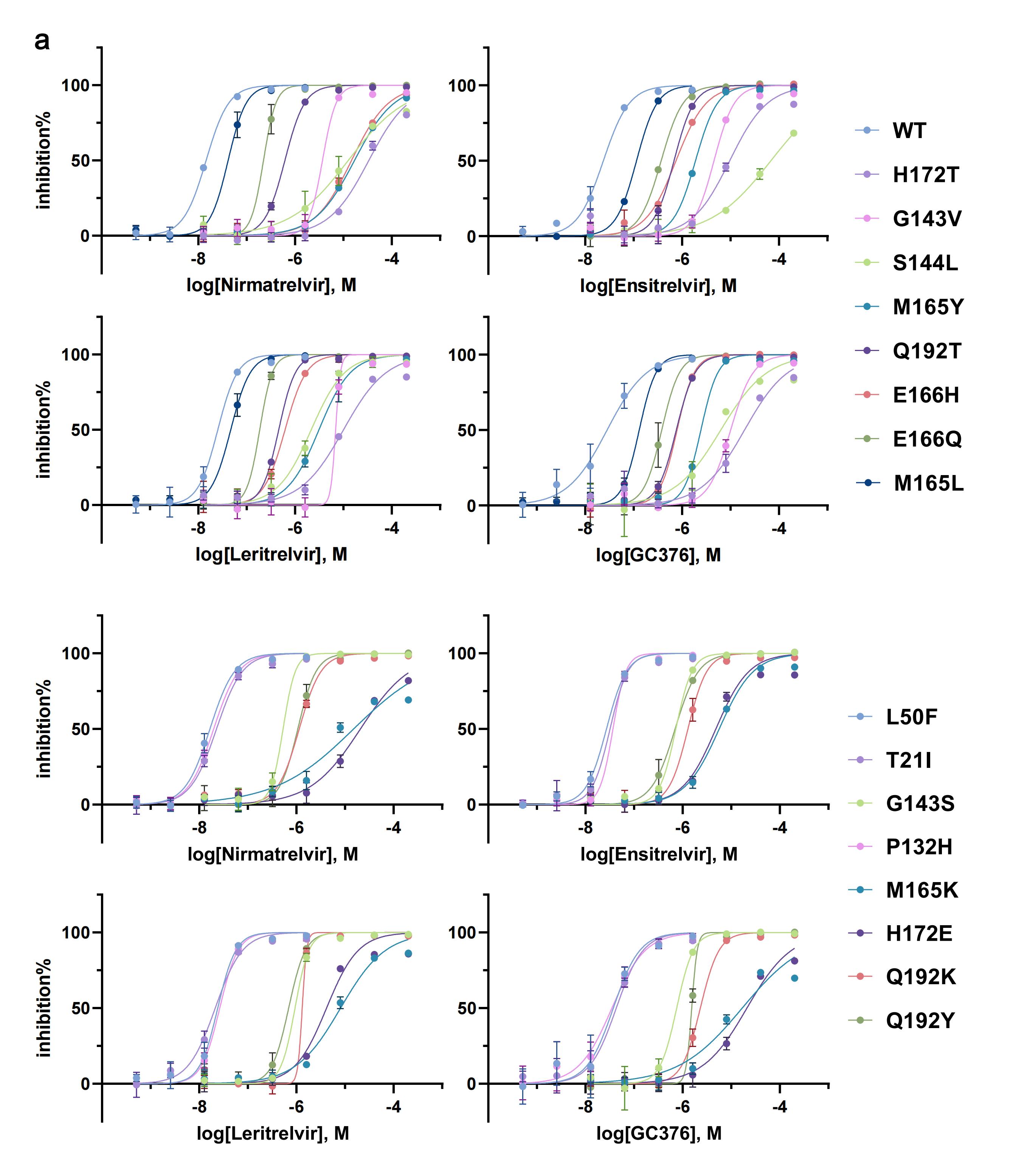

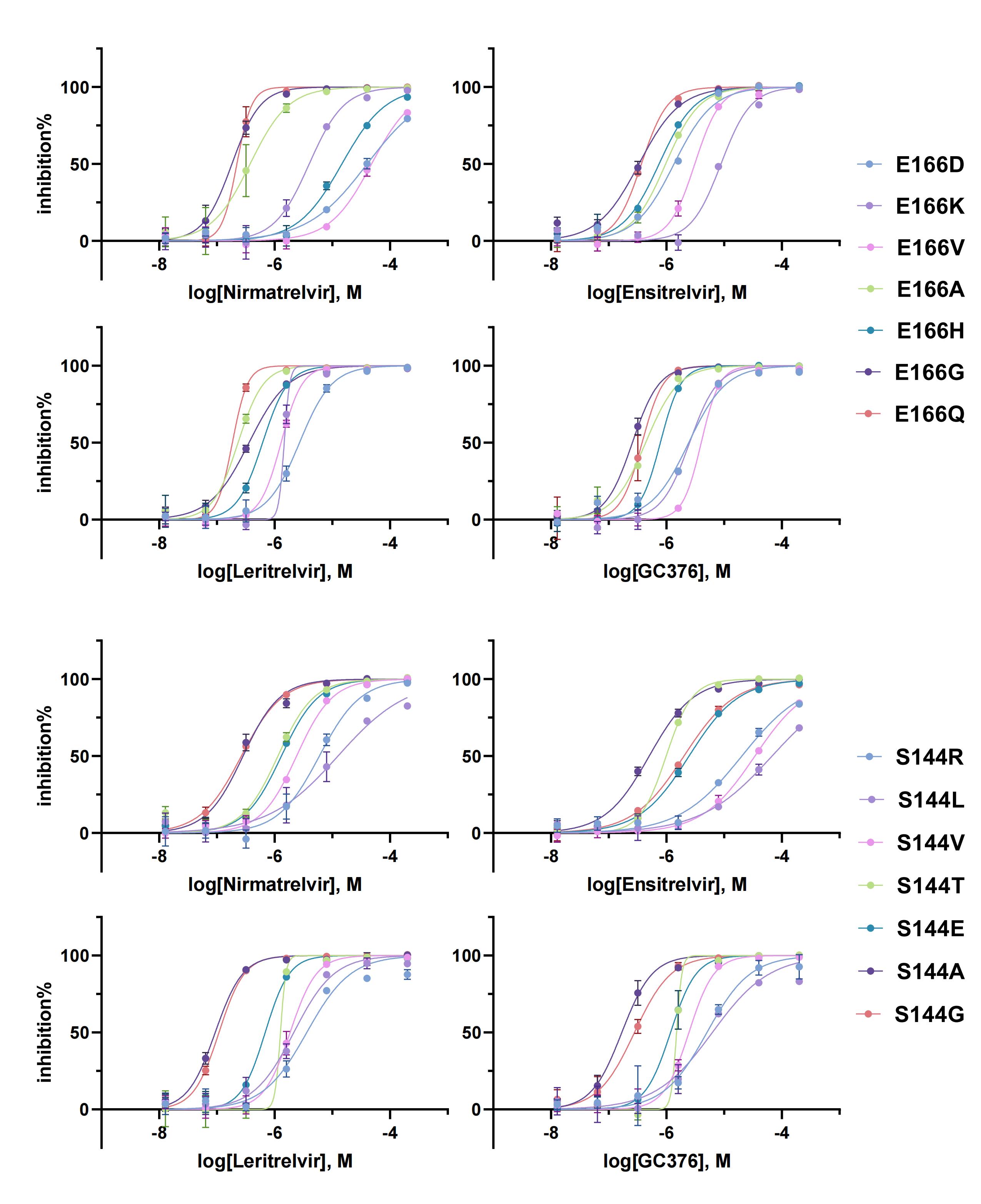


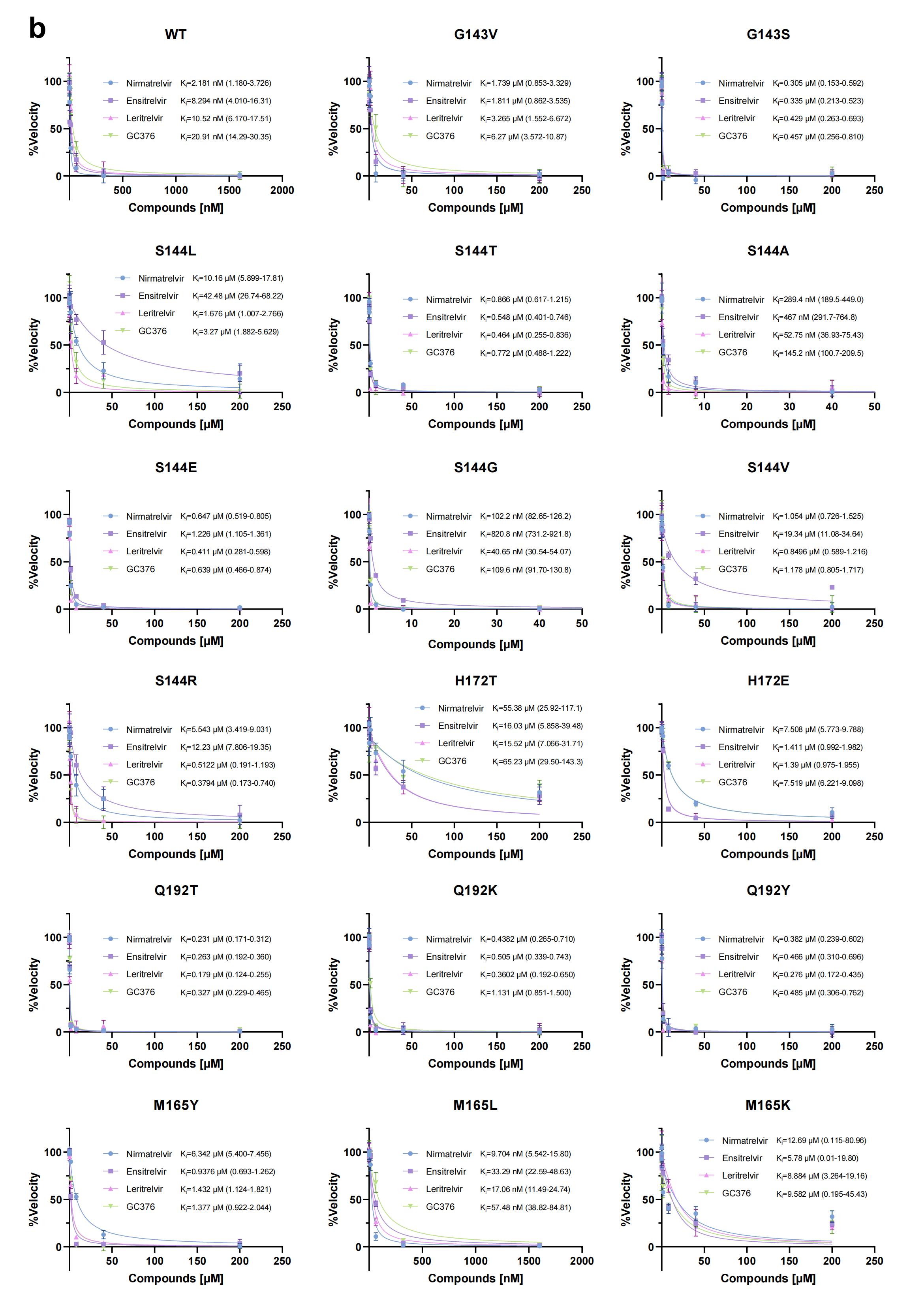

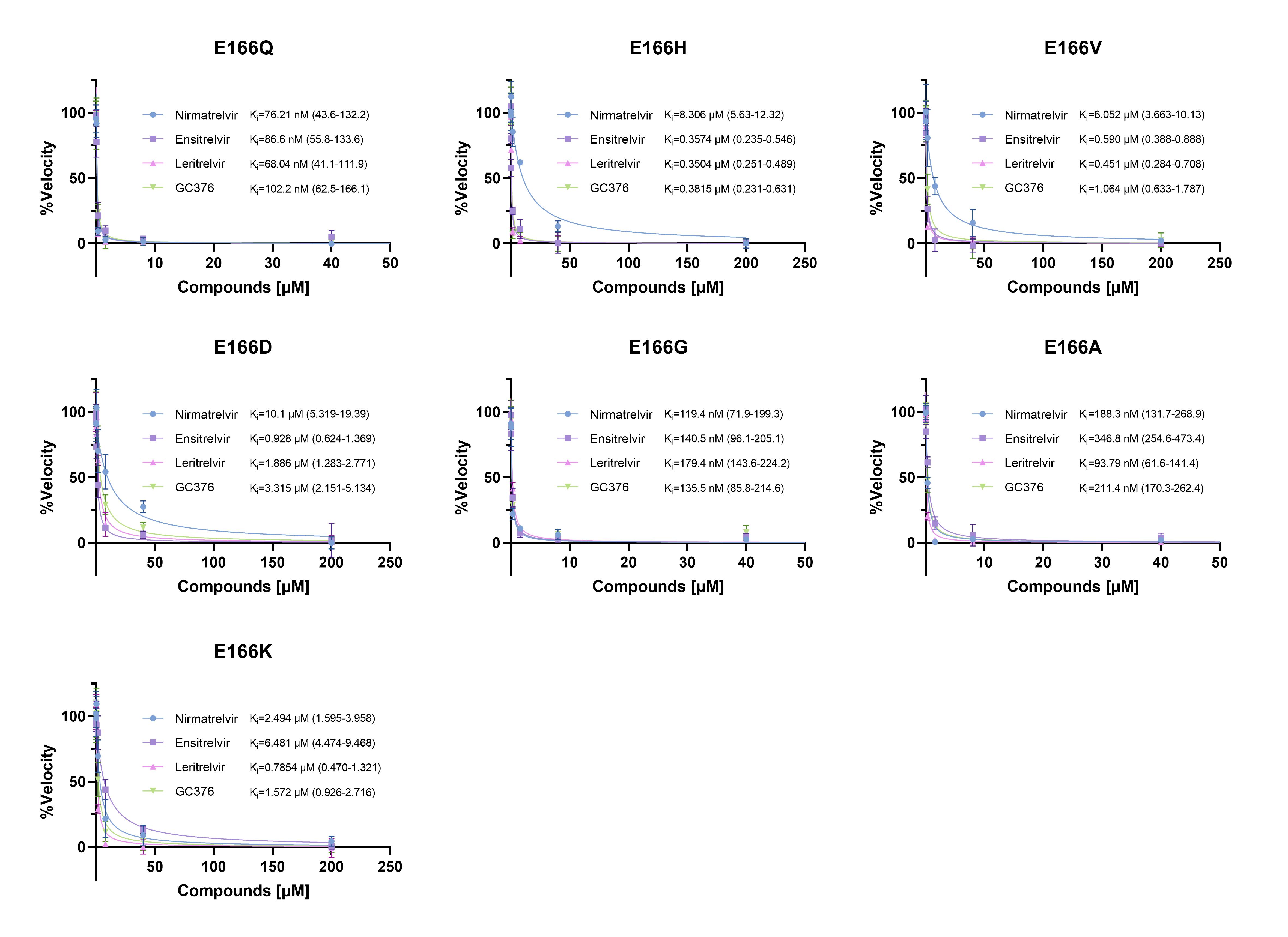


**
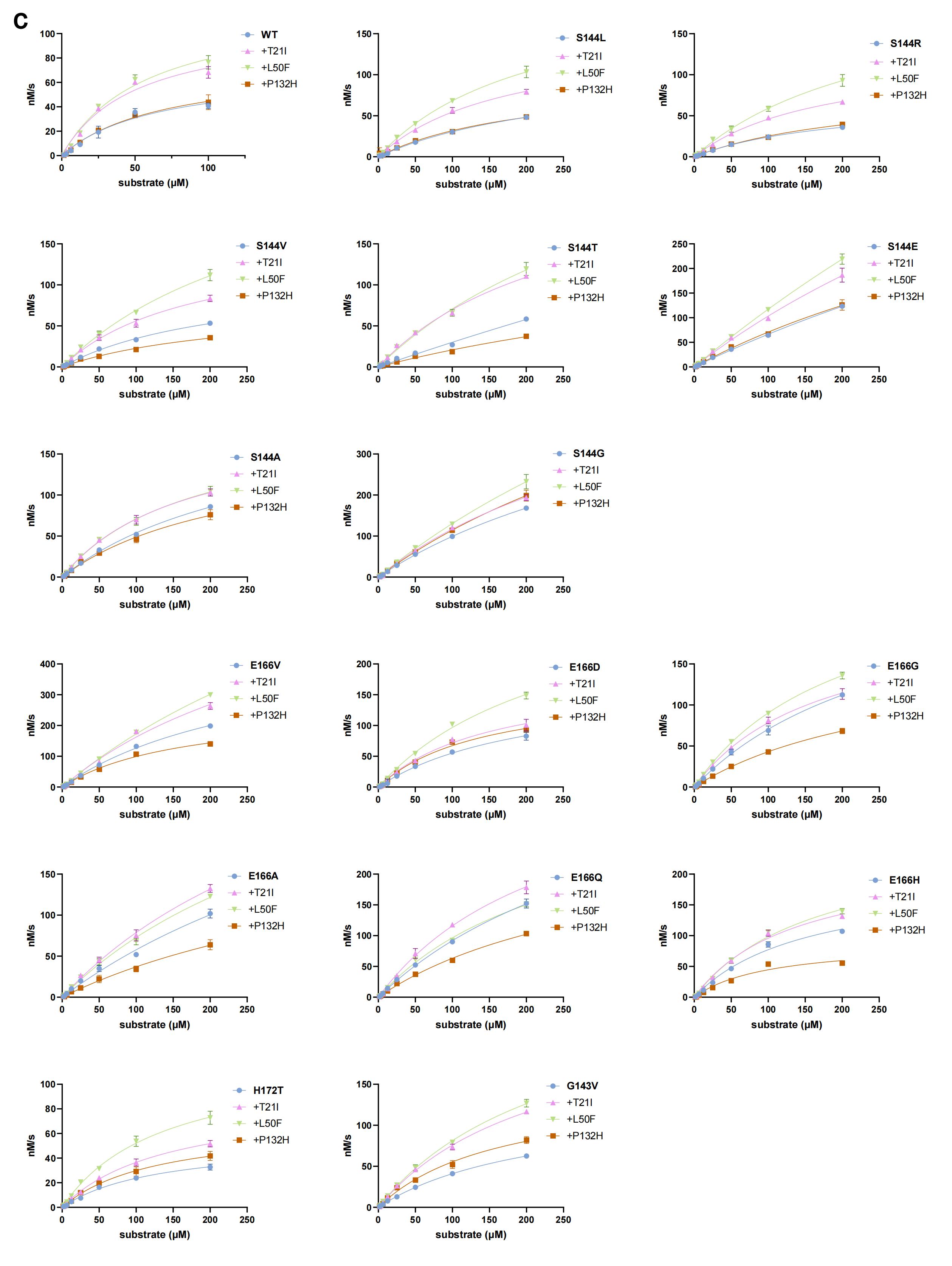

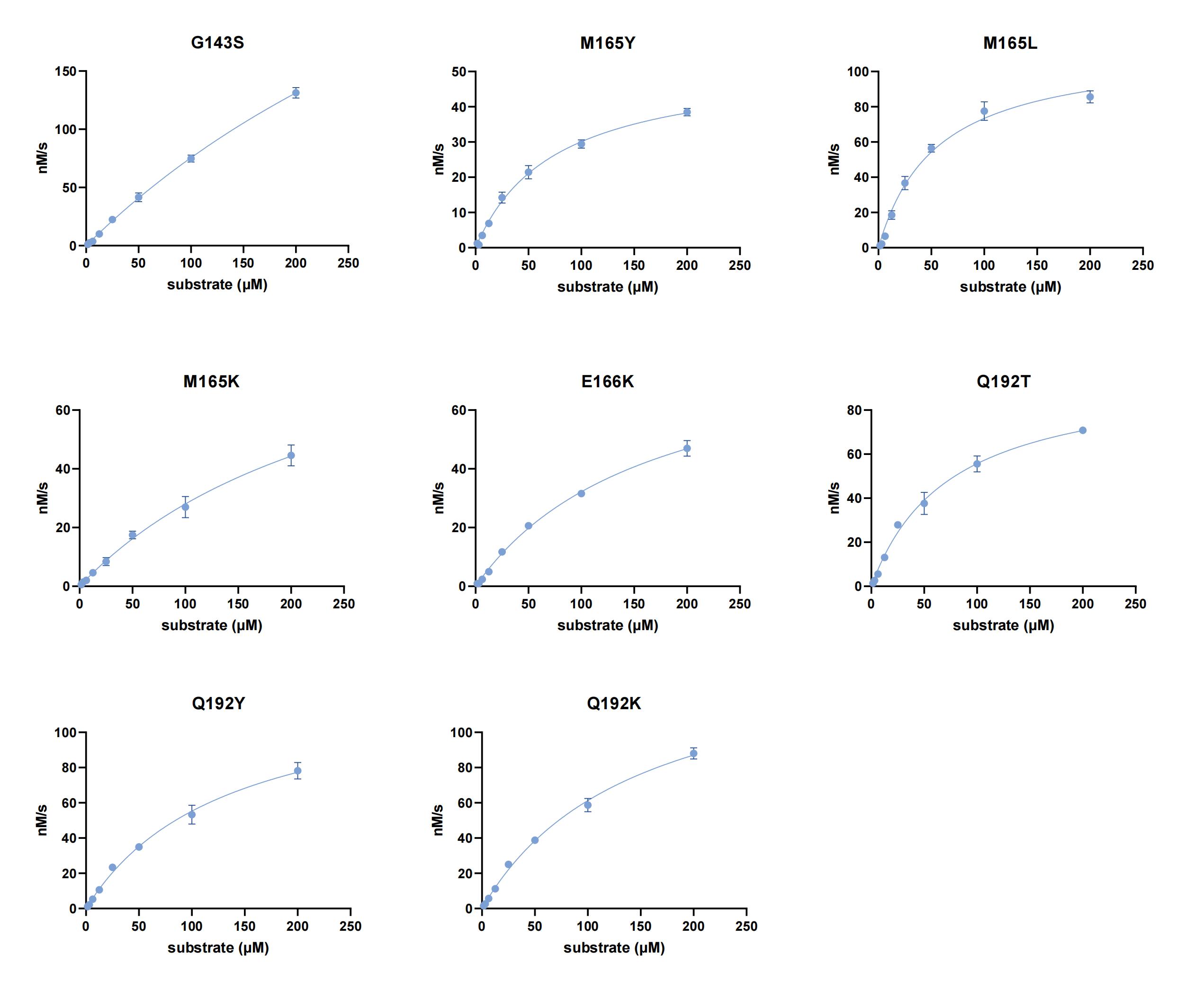
**

**Fig. S2 Characterization of drug inhibition and enzymatic activity of M^pro^ mutants.** **a** The IC_50_ values of nirmatrelvir, ensitrelvir, leritrelvir, and GC376 were determined based on dose–response curves using nonlinear regression. **b** The K_i_ values of four M^pro^ inhibitors were determined based on the initial velocity in the presence of various concentrations of the compounds using Morrison equation (tight inhibition). **c** The K_cat_/K_m_ values of wild-type M^pro^, single, and double mutants were determined based on the initial velocity in the presence of various concentrations of the substrate using Michaelis-Menten model. The above data represent mean ± SEM from 3 experiments.

**Fig. S3**


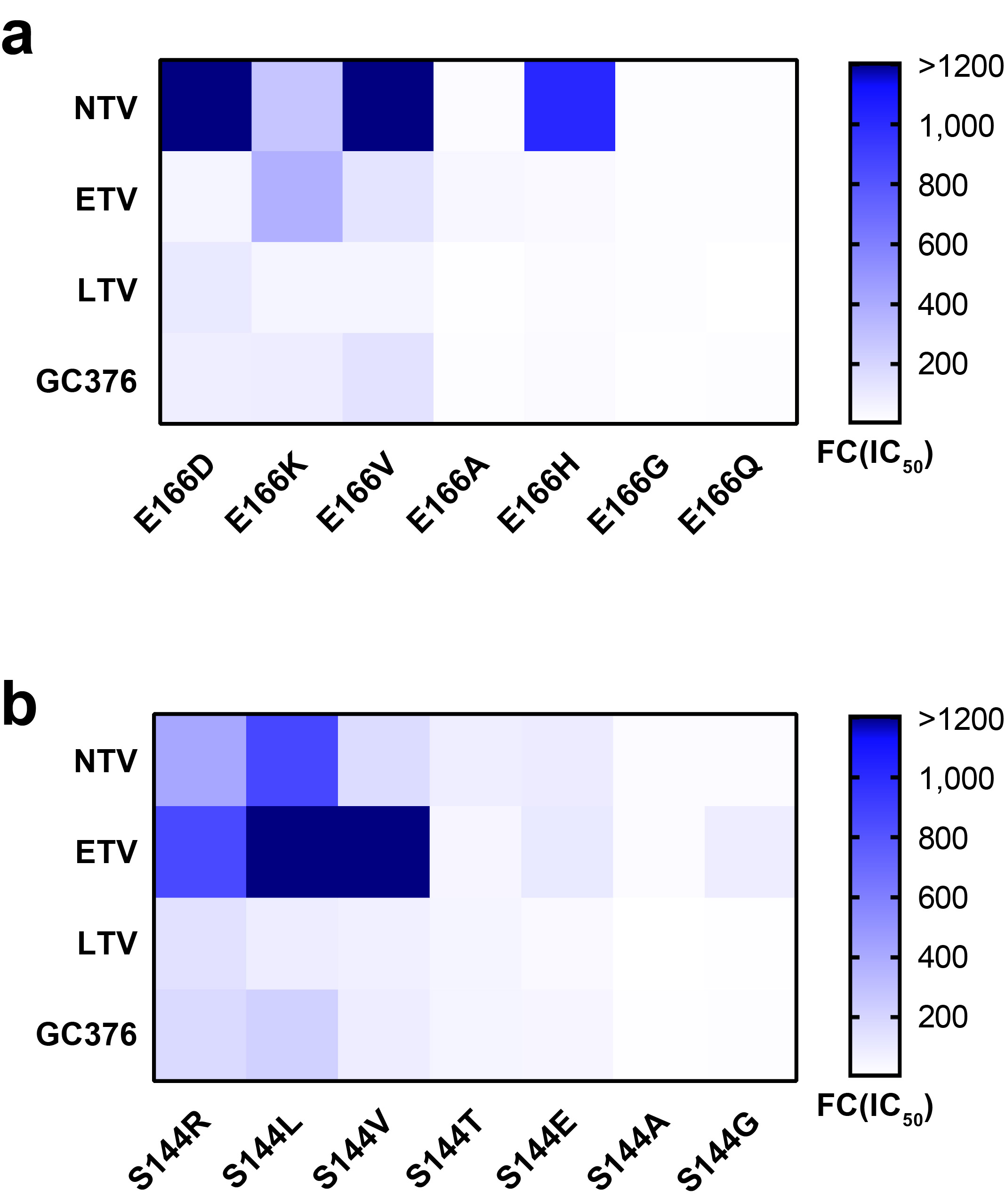


**Fig. S3 Fold-change in IC_50_ of M^pro^ inhibitors against E166 and S144 mutants relative to WT**. Drug inhibition of different types of substitutions at E166 (**a**) and S144 (**b**) were analyzed using a FRET-based enzymatic assay with three replicates.

**Fig. S4**

**
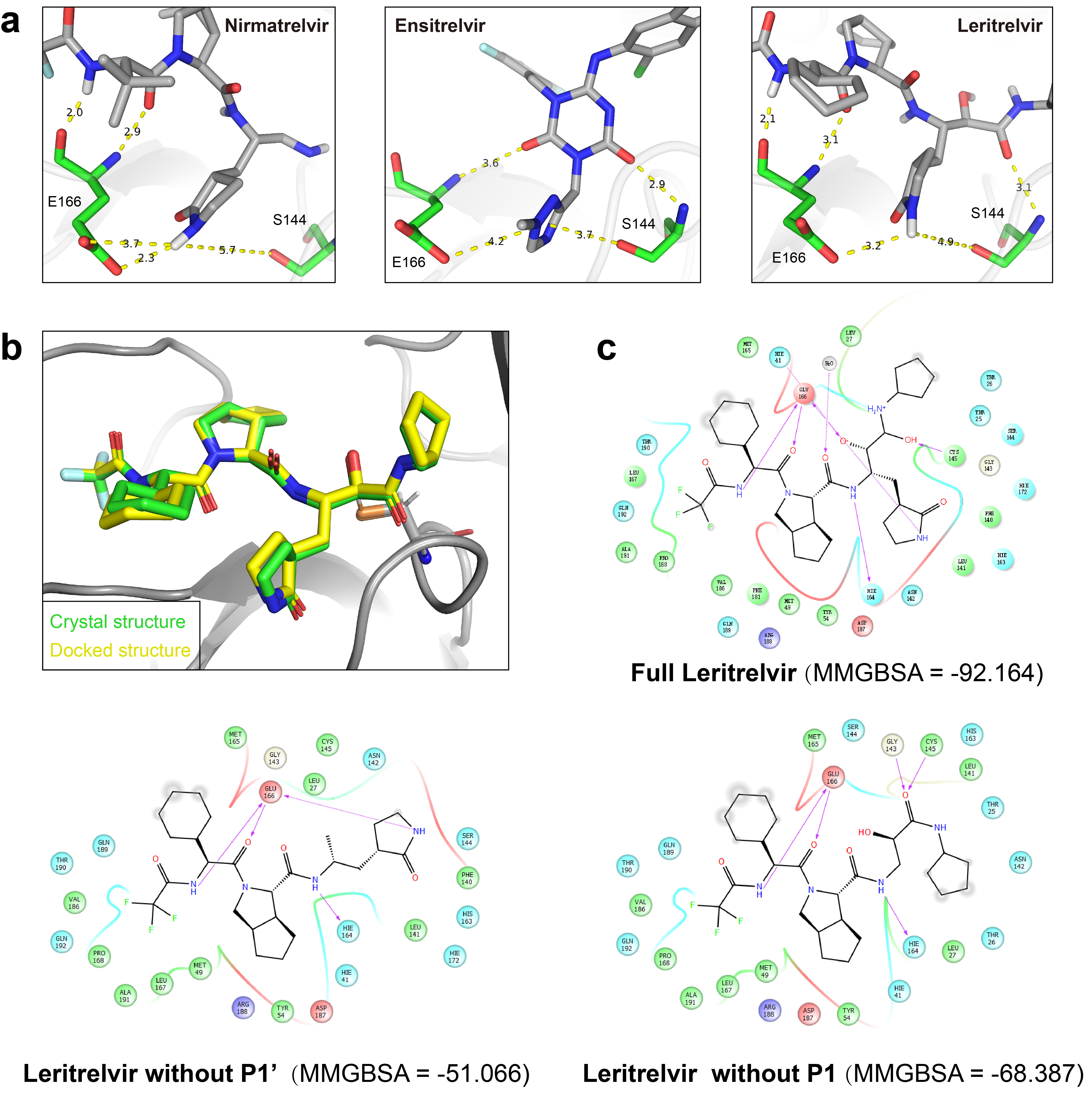
**

**Fig. S4 Structure of SARS-CoV-2 M^pro^ in complex with clinical drug.** **a** Binding poses of nirmatrelvir (PDB code: 7VH8), ensitrelvir (PDB code: 7VU6), and leritrelvir (PDB code: 8IGN) with E166 and S144 in the M^pro^ pocket**.** Hydrogen-bonding interactions are shown as yellow dashed lines and their intermolecular distances are labeled. **b** Comparison of crystal structure and covalently docking structure of M^pro^ in complex with leritrelvir. **c** 2D intermolecular interaction between M^pro^ pocket and intact, P1’-removed and P1-removed leritrelvir. Their binding affinity calculated by the MMGBSA method is labeled below the pictures.

**Fig. S5**





**Fig. S5 FlipGFP assay for SARS-CoV-2 M^pro^ inhibitors.** **a** Bar chart showing the GFP/mCherry fluorescence ratio of wild-type and mutated M^pro^ groups after the treatment of DMSO or 10 μM nirmatrelvir. 293T cells transfected with only FlipGFP plasmid were included as a negative control. Results are from three independent experiments. Error bars represent mean ± standard deviation (SD) **b** The dose-response curve of the GFP/mCherry fluorescence ratio of three E166 mutants treated with nirmatrelvir, ensitrelvir, and leritrelvir. Results are obtained with three replicates. Error bars represent mean ± SD. **c** Images of FlipGFP assay for three E166 mutants. Transfected 293T cells were treated with DMSO or 10 μM M^pro^ inhibitors. The images are representatives of three individual repeats.

**Fig. S6**


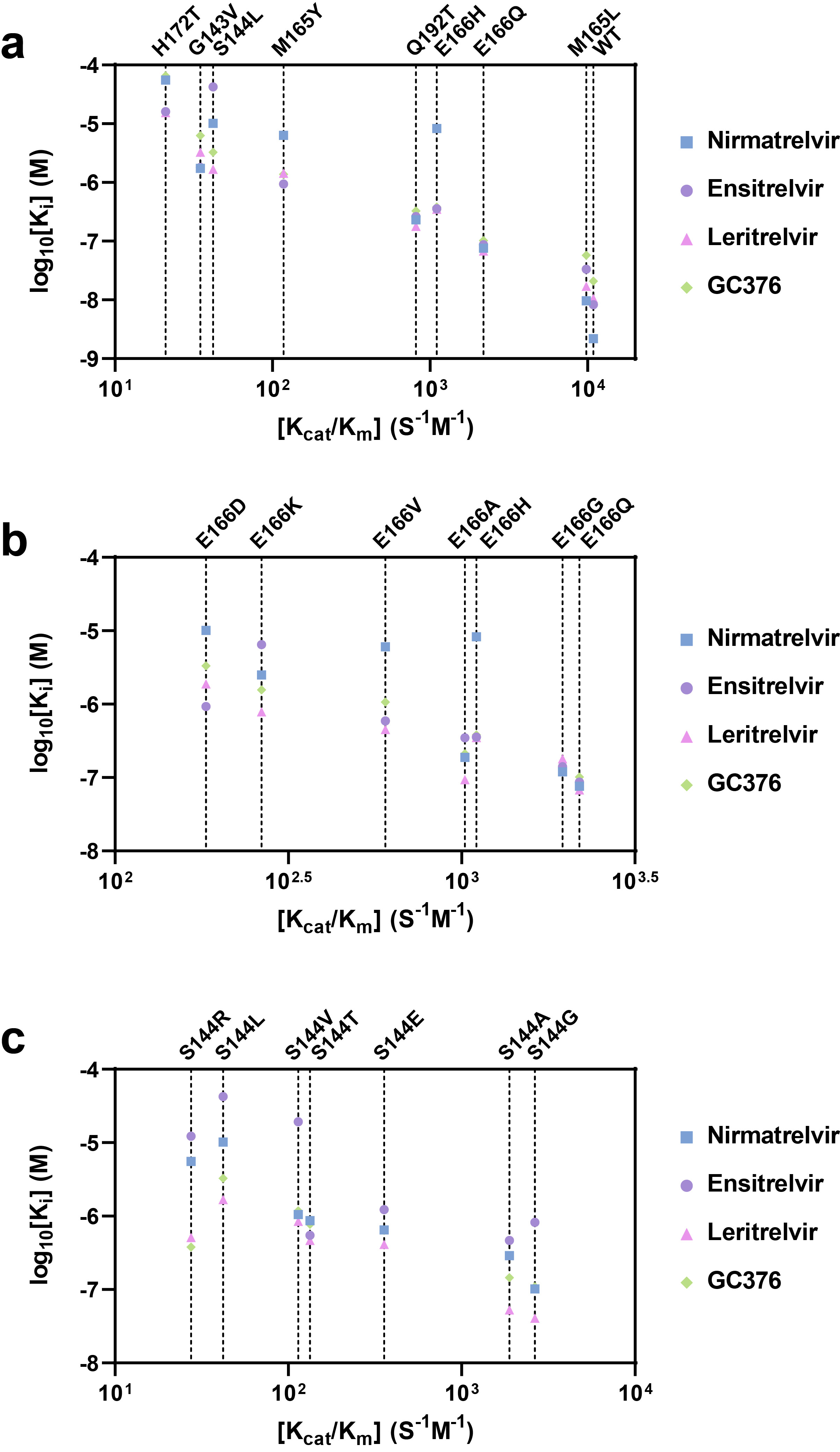


**Fig. S6 Evaluating the association between resistance and enzymatic activity of M^pro^ mutants.** Dot plot showing the K_cat_/K_m_ and K_i_ values of wild-type M^pro^ and single mutants (**a**), E166 (**b**), and S144 (**c**) mutants. These values were determined in three replicates as shown in Supplementary Table S1.

**Fig. S7**


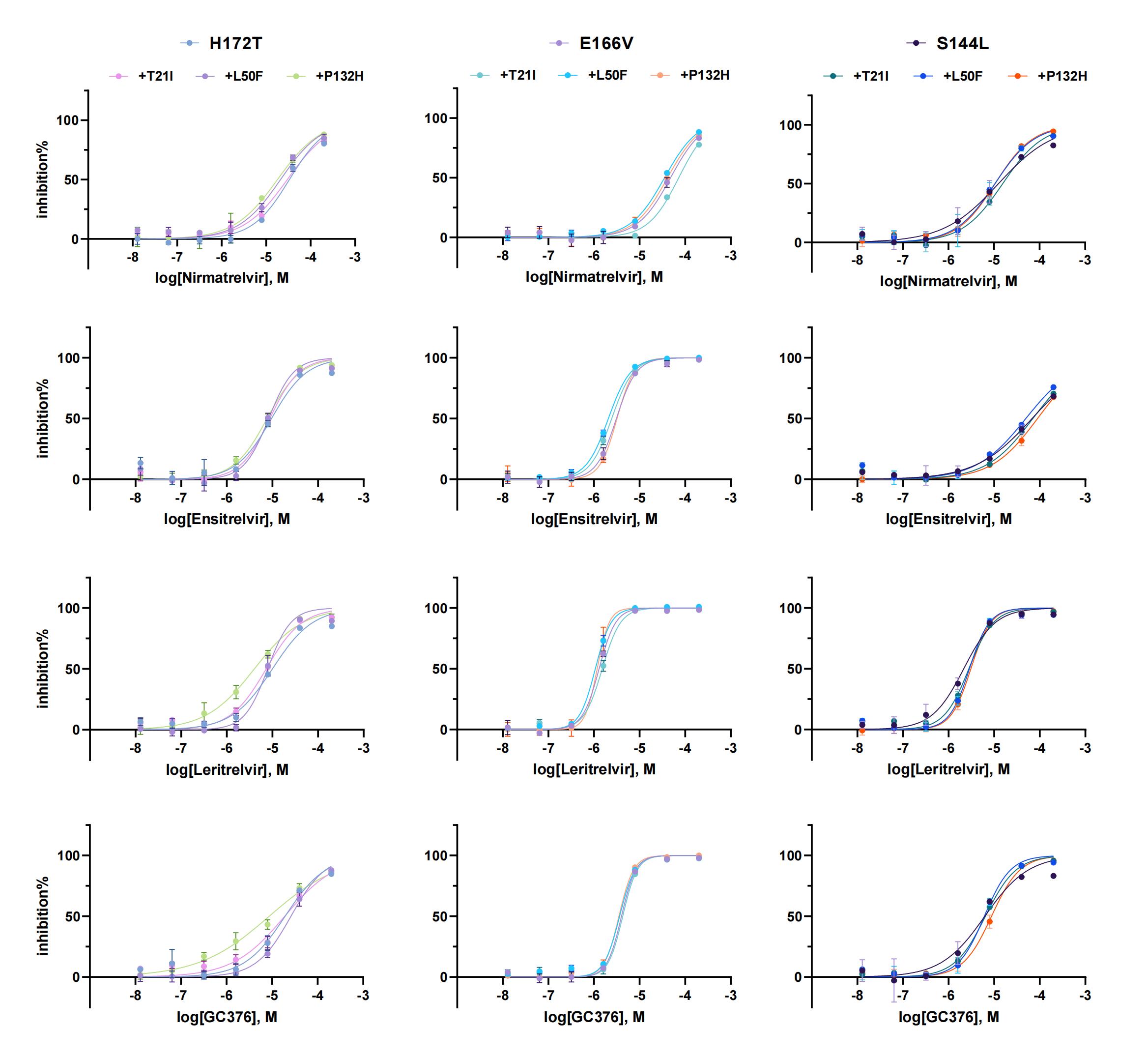


**Fig. S7 Resistance of double mutants to M^pro^ inhibitors.** Three types of substitutions, T21I, L50F, and P132H, were introduced to M^pro^ mutants, including H172T, E166V, and S144L. The IC_50_ values of nirmatrelvir, ensitrelvir, leritrelvir, and GC376 were determined based on dose–response curves using nonlinear regression. The data represent mean ± SEM from 3 experiments.

**Fig. S8**


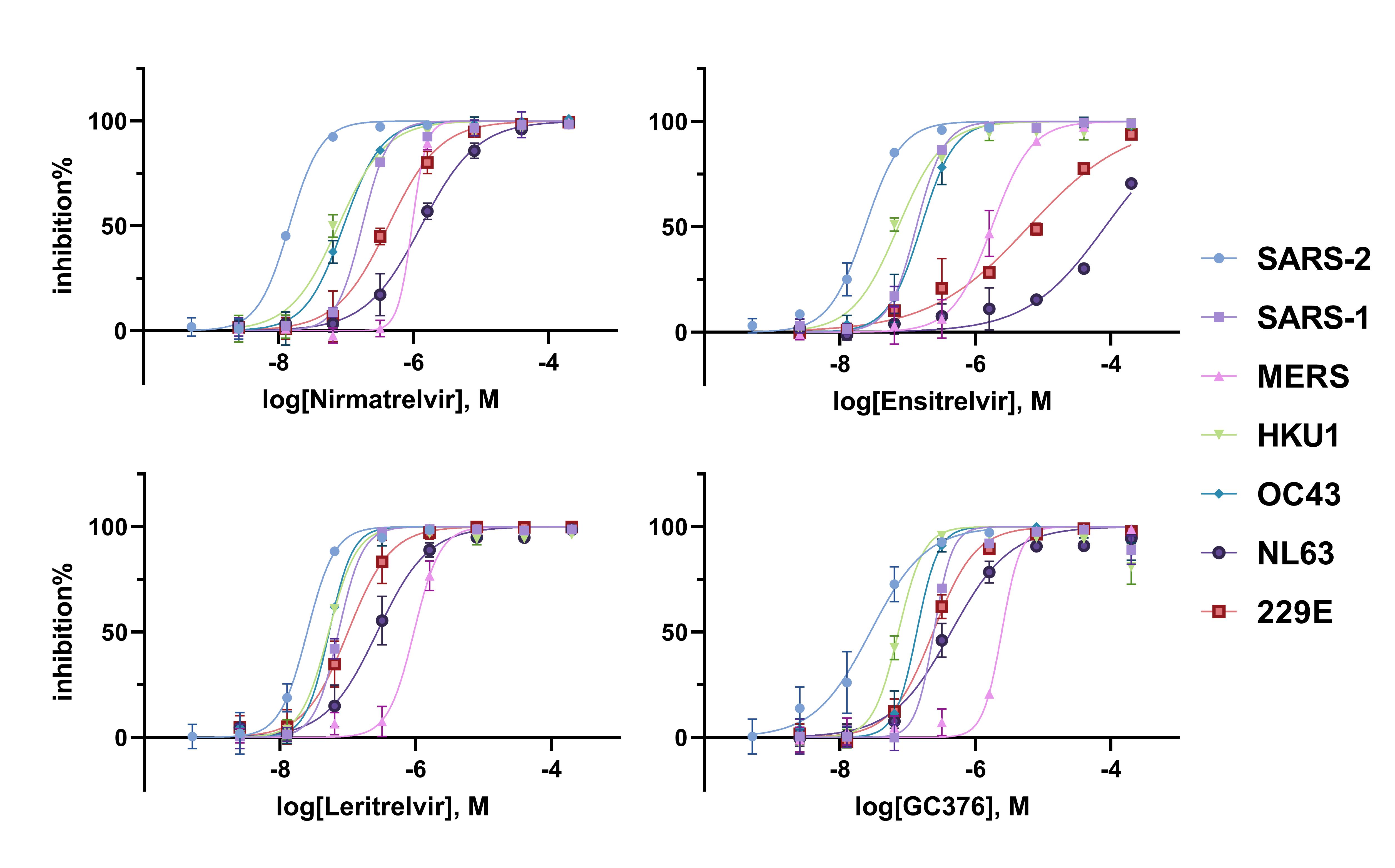


**Fig. S8 Drug inhibition of other coronaviral M^pro^.** The IC_50_ values of nirmatrelvir, ensitrelvir, leritrelvir, and GC376 were determined based on dose–response curves using nonlinear regression. The data represent mean ± SEM from 3 experiments.

**Fig. S9**


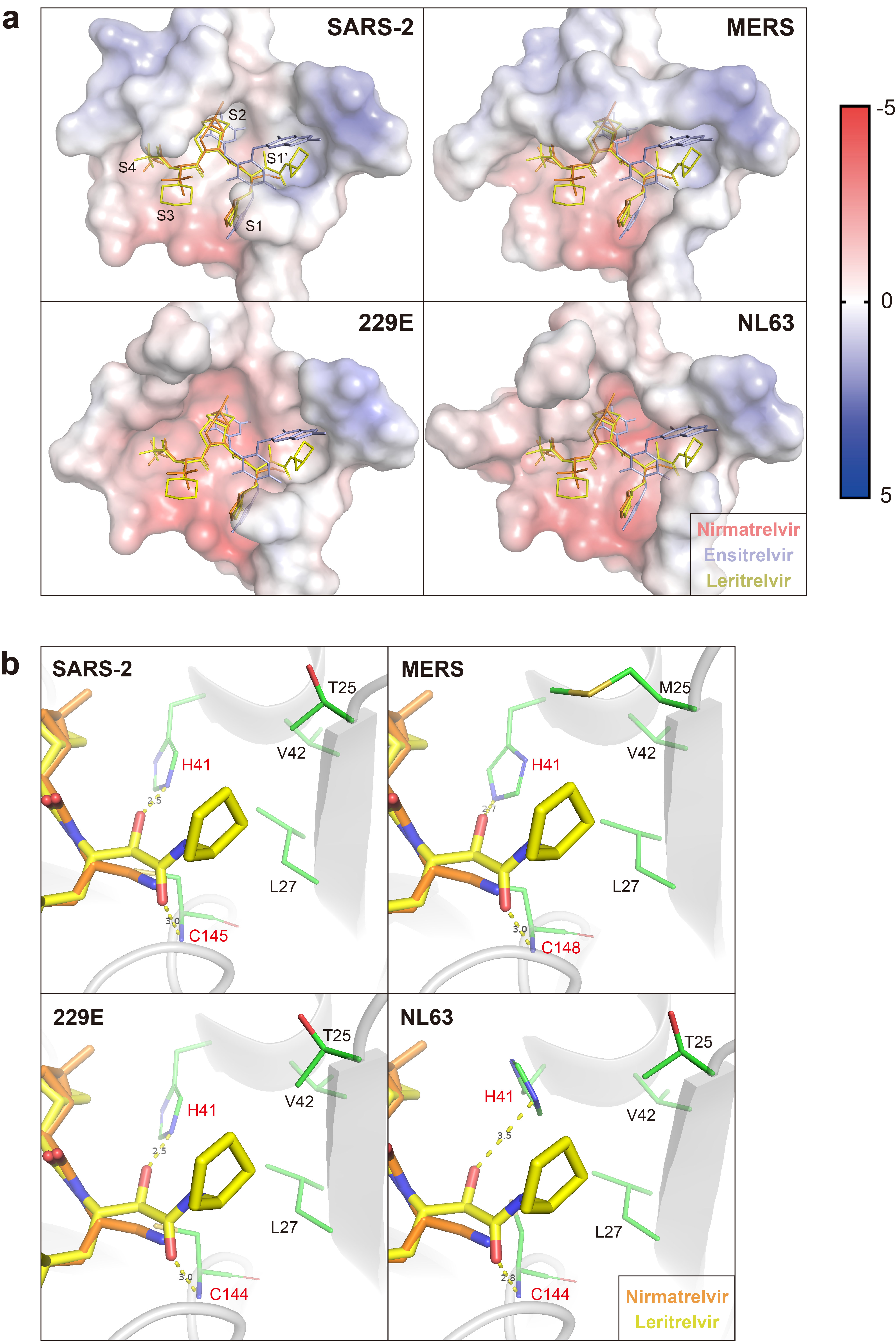


**
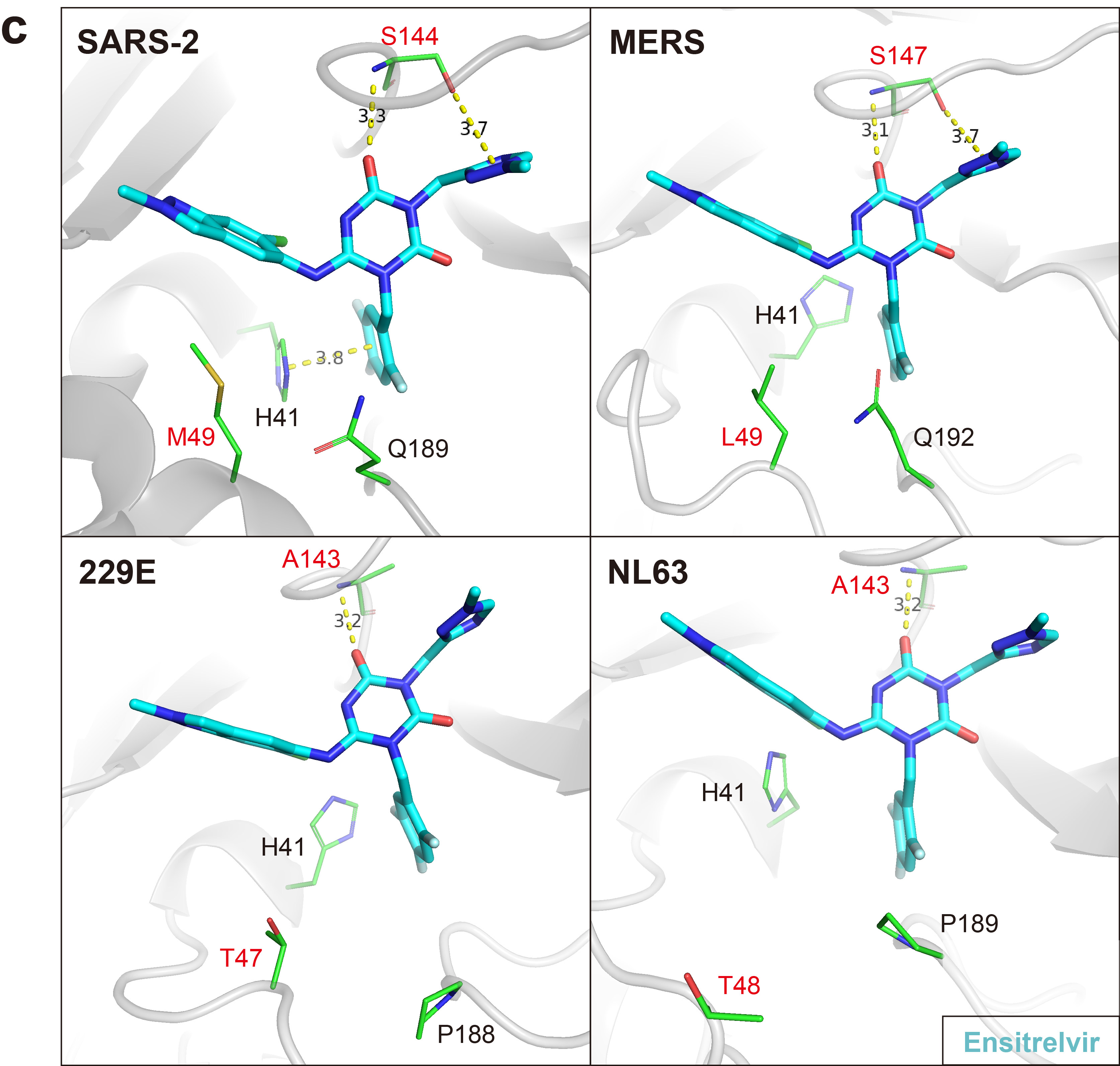
**

**Fig. S9 Structure of coronaviral M^pro^ in complex with clinical drug.** **a** Vacuum electrostatics models showing the highly charged pockets (negative in red and positive in blue) of SARS-2 (PDB code: 8IGN), MERS (PDB code: 4RSP), 229E (PDB code: 2ZU2), and NL63 (PDB code: 3TLO). The inhibitor-binding subsites (S1’, S1, S2, S3 and S4) are labeled in SARS-2 M^pro^. **b** Binding mode at S1’ subsite of nirmatrelvir and leritrelvir in different coronaviral M^pro^ pockets**.** Hydrogen-bonding interactions are shown as yellow dashed lines and pivotal inhibitor-binding residues are colored in red. **c** Binding mode of ensitrelvir in different coronaviral M^pro^ pockets**.** Hydrogen-bonding and π–π stacking interactions are shown as yellow dashed lines and pivotal inhibitor-binding residues that are different among coronaviruses are colored in red.

**Supplementary Table S1**

| **Enzymatic characterization and drug inhibition of SARS-CoV-2 M^pro^ mutants** | | | | | | |
| --- | --- | --- | --- | --- | --- | --- |
| **Mpro mutants** | **Occurrence** | **Enzymatic parameters** | **Nirmatrelvir**  **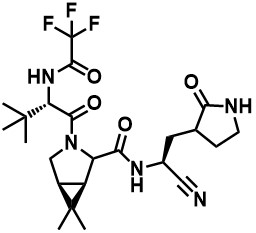** | **Ensitrelvir**  **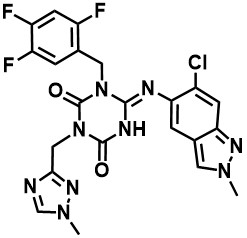** | **Leritrelvir**  **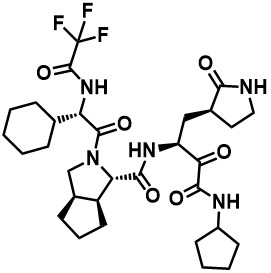** | **GC376**  **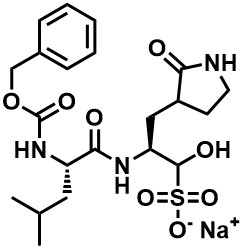** |
| WT |  | M^pro^ con. = 100 nM  K_m_ **=** 65.49 μM  V_max_ **=** 71.15 nM/s  K_cat_ **=** 0.7115 S^-1^  K_cat_/K_m_ = 10864.25 S^-1^M^-1^ | IC_50_ = 14.46 ± 0.64 nM K_i_ = 2.181 ± 0.65 nM | IC_50_ = 23.7 ± 1.81 nM K_i_ = 8.294 ± 3.14 nM | IC_50_ = 25.25 ± 2.12 nM K_i_ = 10.52 ± 2.89 nM | IC_50_ = 28.23 ± 4.57 nM K_i_ = 20.91 ± 4.1 nM |
| L50F | 5236 | M^pro^ con. = 100 nM  K_m_ **=** 58.97 μM  V_max_ **=** 125.9 nM/s  K_cat_ **=** 1.259 S^-1^  K_cat_/K_m_ = 21349.84 S^-1^M^-1^ | IC_50_ = 16.59 ± 1.03 nM | IC_50_ = 27.02 ± 1.56 nM | IC_50_ = 24.01 ± 2.28 nM | IC_50_ = 37.34 ± 7.17 nM |
| T21I | 20100 | M^pro^ con. = 100 nM  K_m_ **=** 51.09 μM  V_max_ **=** 108.7 nM/s  K_cat_ **=** 1.087 S^-1^  K_cat_/K_m_ **=** 21276.18 S^-1^M^-1^ | IC_50_ = 22.66 ± 1.61 nM | IC_50_ = 32.0 ± 2.94 nM | IC_50_ = 21.34 ± 1.86 nM | IC_50_ = 42.96 ± 4.90 nM |
| P132H | 8107451 | M^pro^ con. = 100 nM  K_m_ **=** 70.52 μM  V_max_ **=** 75.91 nM/s  K_cat_ **=** 0.7591 S^-1^  K_cat_/K_m_ **=** 10764.32 S^-1^M^-1^ | IC_50_ = 19.82 ± 1.10 nM | IC_50_ = 36.75 ± 2.77 nM | IC_50_ = 26.41 ± 1.92 nM | IC_50_ = 34.99 ± 5.76 nM |
| G143V | 46 | M^pro^ con. = 17.13 μM  K_m_ **=** 220.7 μM  V_max_ **=** 131.5 nM/s  K_cat_ **=** 0.007678 S^-1^  K_cat_/K_m_ **=** 34.79 S^-1^M^-1^ | IC_50_ = 3.681 ± 0.41 μM Ki = 1.739 ± 0.63 μM | IC_50_ = 4.541 ± 0.34 μM Ki = 1.811 ± 0.68 μM | IC_50_ = 6.877 ± 0.68 μM Ki = 3.265 ± 1.31 μM | IC_50_ = 10.01 ± 0.71 μM Ki = 6.27 ± 1.86 μM |
| G143V + L50F |  | M^pro^ con. = 17.13 μM  K_m_ **=** 227.1 μM  V_max_ **=** 247.8 nM/s  K_cat_ **=** 0.01447 S^-1^  K_cat_/K_m_ **=** 63.72 S^-1^M^-1^ |  |  |  |  |
| G143V + T21I |  | M^pro^ con. = 17.13 μM  K_m_ **=** 248.2 μM  V_max_ **=** 283 nM/s  K_cat_ **=** 0.01652 S^-1^  K_cat_/K_m_ **=** 66.56 S^-1^M^-1^ |  |  |  |  |
| G143V + P132H |  | M^pro^ con. = 17.13 μM  K_m_ **=** 165.9 μM  V_max_ **=** 147.3 nM/s  K_cat_ **=** 0.0086 S^-1^  K_cat_/K_m_ **=** 51.84 S^-1^M^-1^ |  |  |  |  |
| G143S | 24 | M^pro^ con. = 1.995 μM  K_m_ **=** 561.7 μM  V_max_ **=** 499.7 nM/s  K_cat_ **=** 0.2505 S^-1^  K_cat_/K_m_ **=** 445.97 S^-1^M^-1^ | IC_50_ = 0.530 ± 0.06 μM Ki = 0.3054 ± 0.11 μM | IC_50_ = 0.722 ± 0.04 μM Ki = 0.3354 ± 0.08 μM | IC_50_ = 0.934 ± 0.05 μM Ki = 0.429 ± 0.11 μM | IC_50_ = 0.752 ± 0.07 μM Ki = 0.457 ± 0.14 μM |
| S144L | 66 | M^pro^ con. = 9.985 μM  K_m_ **=** 270.3 μM  V_max_ **=** 113.3 nM/s  K_cat_ **=** 0.01134 S^-1^  K_cat_/K_m_ **=** 41.95 S^-1^M^-1^ | IC_50_ = 12.53 ± 2.1 μM Ki = 10.16 ± 3.04 μM | IC_50_ = 67.73 ± 7.20 μM Ki = 42.48 ± 10.58 μM | IC_50_ = 2.153 ± 0.21 μM Ki = 1.676 ± 0.45 μM | IC_50_ = 6.258 ± 1.28 μM Ki = 3.27 ± 0.96 μM |
| S144L + L50F |  | M^pro^ con. = 9.985 μM  K_m_ **=** 173.9 μM  V_max_ **=** 149.4 nM/s  K_cat_ **=** 0.01496 S^-1^  K_cat_/K_m_ **=** 86.03 S^-1^M^-1^ | IC_50_ = 10.49 ± 1.4 μM | IC_50_ = 49.44 ± 5.85 μM | IC_50_ = 2.875 ± 0.21 μM | IC_50_ = 6.103 ± 0.47 μM |
| S144L + T21I |  | M^pro^ con. = 9.985 μM  K_m_ **=** 213.2 μM  V_max_ **=** 213.7 nM/s  K_cat_ **=** 0.0214 S^-1^  K_cat_/K_m_ **=** 100.38 S^-1^M^-1^ | IC_50_ = 15.54 ± 1.34 μM | IC_50_ = 70.6 ± 6.3 μM | IC_50_ = 2.791 ± 0.21 μM | IC_50_ = 6.444 ± 0.28 μM |
| S144L + P132H |  | M^pro^ con. = 9.985 μM  K_m_ **=** 209.7 μM  V_max_ **=** 98.66 nM/s  K_cat_ **=** 0.00988 S^-1^  K_cat_/K_m_ **=** 47.11 S^-1^M^-1^ | IC_50_ = 10.56 ± 0.86 μM | IC_50_ = 89.3 ± 4.93 μM | IC_50_ = 3.092 ± 0.2 μM | IC_50_ = 8.763 ± 0.53 μM |
| S144T | 16 | M^pro^ con. = 2.344 μM  K_m_ **=** 2484 μM  V_max_ **=** 776.7 nM/s  K_cat_ **=** 0.3313 S^-1^  K_cat_/K_m_ **=** 133.37 S^-1^M^-1^ | IC_50_ = 1.142 ± 0.11 μM Ki = 0.866 ± 0.15 μM | IC_50_ = 0.991 ± 0.03 μM Ki = 0.548 ± 0.09 μM | IC_50_ = 1.266 μM Ki = 0.464 ± 0.15 μM | IC_50_ = 1.501 μM Ki = 0.772 ± 0.19 μM |
| S144T + L50F |  | M^pro^ con. = 2.344 μM  K_m_ **=** 259 μM  V_max_ **=** 250.9 nM/s  K_cat_ **=** 0.107 S^-1^  K_cat_/K_m_ **=** 413.13 S^-1^M^-1^ |  |  |  |  |
| S144T + T21I |  | M^pro^ con. = 2.344 μM  K_m_ **=** 411.4 μM  V_max_ **=** 361.2 nM/s  K_cat_ **=** 0.1541 S^-1^  K_cat_/K_m_ **=** 374.57 S^-1^M^-1^ |  |  |  |  |
| S144T + P132H |  | M^pro^ con. = 2.344 μM  K_m_ **=** 690.4 μM  V_max_ **=** 164.1 nM/s  K_cat_ **=** 0.07003 S^-1^  K_cat_/K_m_ **=** 101.43 S^-1^M^-1^ |  |  |  |  |
| S144A | 17 | M^pro^ con. = 388.8 nM  K_m_ **=** 279.1 μM  V_max_ **=** 204.5 nM/s  K_cat_ **=** 0.526 S^-1^  K_cat_/K_m_ **=** 1884.63 S^-1^M^-1^ | IC_50_ = 0.284 ± 0.02 μM Ki = 289.4 ± 66.3 nM | IC_50_ = 0.504 ± 0.03 μM Ki = 467 ± 121 nM | IC_50_ = 0.094 ± 0.01 μM Ki = 52.75 ± 10.1 nM | IC_50_ = 0.169 ± 0.01 μM Ki = 145.2 ± 27.9 nM |
| S144A + L50F |  | M^pro^ con. = 388.8 nM  K_m_ **=** 165.7 μM  V_max_ **=** 188.4 nM/s  K_cat_ **=** 0.4845 S^-1^  K_cat_/K_m_ **=** 2923.96 S^-1^M^-1^ |  |  |  |  |
| S144A + T21I |  | M^pro^ con. = 388.8 nM  K_m_ **=** 176.8 μM  V_max_ **=** 195.7 nM/s  K_cat_ **=** 0.5033 S^-1^  K_cat_/K_m_ **=** 2846.72 S^-1^M^-1^ |  |  |  |  |
| S144A + P132H |  | M^pro^ con. = 388.8 nM  K_m_ **=** 236.4 μM  V_max_ **=** 164.1 nM/s  K_cat_ **=** 0.4221 S^-1^  K_cat_/K_m_ **=** 1785.53 S^-1^M^-1^ |  |  |  |  |
| S144E | 6 | M^pro^ con. = 2.018 μM  K_m_ **=** 1169 μM  V_max_ **=** 841.9 nM/s  K_cat_ **=** 0.4172 S^-1^  K_cat_/K_m_ **=** 356.89 S^-1^M^-1^ | IC_50_ = 1.312 ± 0.07 μM Ki = 0.647 ± 0.07 μM | IC_50_ = 2.468 ± 0.1 μM Ki = 1.23 ± 0.07 μM | IC_50_ = 0.687 ± 0.05 μM Ki = 0.411 ± 0.08 μM | IC_50_ = 1.18 ± 0.07 μM Ki = 0.639 ± 0.1 μM |
| S144E + L50F |  | M^pro^ con. = 2.018 μM  K_m_ **=** 788.6 μM  V_max_ **=** 917.1 nM/s  K_cat_ **=** 0.4545 S^-1^  K_cat_/K_m_ **=** 576.34 S^-1^M^-1^ |  |  |  |  |
| S144E + T21I |  | M^pro^ con. = 2.018 μM  K_m_ **=** 1318 μM  V_max_ **=** 1661 nM/s  K_cat_ **=** 0.8228 S^-1^  K_cat_/K_m_ **=** 624.28 S^-1^M^-1^ |  |  |  |  |
| S144E + P132H |  | M^pro^ con. = 2.018 μM  K_m_ **=** 653.7 μM  V_max_ **=** 533.4 nM/s  K_cat_ **=** 0.2643 S^-1^  K_cat_/K_m_ **=** 404.31 S^-1^M^-1^ |  |  |  |  |
| S144G | 1 | M^pro^ con. = 456.3 nM  K_m_ **=** 460.1 μM  V_max_ **=** 555.4 nM/s  K_cat_ **=** 1.217 S^-1^  K_cat_/K_m_ **=** 2645.08 S^-1^M^-1^ | IC_50_ = 0.266 ± 0.01 μM Ki = 102.2 ± 11.3 nM | IC_50_ = 2.016 ± 0.08 μM Ki = 820.8 ± 48.7 nM | IC_50_ = 0.109 ± 0.01 μM Ki = 40.65 ± 6.1 nM | IC_50_ = 0.28 ± 0.02 μM Ki = 109.6 ± 10.2 nM |
| S144G + L50F |  | M^pro^ con. = 456.3 nM  K_m_ **=** 403.2 μM  V_max_ **=** 587.9 nM/s  K_cat_ **=** 1.288 S^-1^  K_cat_/K_m_ **=** 3194.44 S^-1^M^-1^ |  |  |  |  |
| S144G + T21I |  | M^pro^ con. = 456.3 nM  K_m_ **=** 723 μM  V_max_ **=** 1072 nM/s  K_cat_ **=** 2.35 S^-1^  K_cat_/K_m_ **=** 3250.35 S^-1^M^-1^ |  |  |  |  |
| S144G + P132H |  | M^pro^ con. = 456.3 nM  K_m_ **=** 566.7 μM  V_max_ **=** 761.7 nM/s  K_cat_ **=** 1.669 S^-1^  K_cat_/K_m_ **=** 2945.12 S^-1^M^-1^ |  |  |  |  |
| S144V | 3 | M^pro^ con. = 4.392 μM  K_m_ **=** 223.9 μM  V_max_ **=** 112.2 nM/s  K_cat_ **=** 0.02554 S^-1^  K_cat_/K_m_ **=** 114.07 S^-1^M^-1^ | IC_50_ = 2.406 ± 0.12 μM Ki = 1.054 ± 0.2 μM | IC_50_ = 34.18 ± 1.79 μM Ki = 19.34 ± 6.01 μM | IC_50_ = 1.856 ± 0.12 μM Ki = 0.85 ± 0.16 μM | IC_50_ = 2.46 ± 0.17 μM Ki = 1.178 ± 0.23 μM |
| S144V + L50F |  | M^pro^ con. = 4.392 μM  K_m_ **=** 179 μM  V_max_ **=** 156.5 nM/s  K_cat_ **=** 0.03564 S^-1^  K_cat_/K_m_ **=** 199.11 S^-1^M^-1^ |  |  |  |  |
| S144V + T21I |  | M^pro^ con. = 4.392 μM  K_m_ **=** 311.8 μM  V_max_ **=** 284.4 nM/s  K_cat_ **=** 0.06477 S^-1^  K_cat_/K_m_ **=** 207.73 S^-1^M^-1^ |  |  |  |  |
| S144V + P132H |  | M^pro^ con. = 4.392 μM  K_m_ **=** 241 μM  V_max_ **=** 77.44 nM/s  K_cat_ **=** 0.01763 S^-1^  K_cat_/K_m_ **=** 73.15 S^-1^M^-1^ |  |  |  |  |
| S144R | 2 | M^pro^ con. = 13.314 μM  K_m_ **=** 194.3 μM  V_max_ **=** 71 nM/s  K_cat_ **=** 0.005333 S^-1^  K_cat_/K_m_ **=** 27.45 S^-1^M^-1^ | IC_50_ = 5.971 ± 0.61 μM Ki = 5.543 ± 1.43 μM | IC_50_ = 20.28 ± 2.03 μM Ki = 12.23 ± 2.94 μM | IC_50_ = 3.612 ± 0.53 μM Ki = 0.512 ± 0.26 μM | IC_50_ = 5.043 ± 0.76 μM Ki = 0.379 ± 0.14 μM |
| S144R + L50F |  | M^pro^ con. = 13.314 μM  K_m_ **=** 175.3 μM  V_max_ **=** 126.6 nM/s  K_cat_ **=** 0.009506 S^-1^  K_cat_/K_m_ **=** 54.23 S^-1^M^-1^ |  |  |  |  |
| S144R + T21I |  | M^pro^ con. = 13.314 μM  K_m_ **=** 265.1 μM  V_max_ **=** 215.9 nM/s  K_cat_ **=** 0.01621 S^-1^  K_cat_/K_m_ **=** 61.15 S^-1^M^-1^ |  |  |  |  |
| S144R + P132H |  | M^pro^ con. = 13.314 μM  K_m_ **=** 238.1 μM  V_max_ **=** 85.48 nM/s  K_cat_ **=** 0.006421 S^-1^  K_cat_/K_m_ **=** 26.97 S^-1^M^-1^ |  |  |  |  |
| S144K |  | M^pro^ con. = 16.446 μM  K_m_ **=** 42.44 μM  V_max_ **=** 1.602 nM/s  K_cat_ **=** 0.00009742 S^-1^  K_cat_/K_m_ **=** 2.30 S^-1^M^-1^ | IC_50_ > 200μM | | | |
| S144P |  | M^pro^ con. = 14.793 μM  K_m_ **=** 129.3 μM  V_max_ **=** 8.617 nM/s  K_cat_ **=** 0.0005825 S^-1^  K_cat_/K_m_ **=** 4.51 S^-1^M^-1^ | IC_50_ > 200μM | | | |
| H172T | 128 | M^pro^ con. = 19.97 μM  K_m_ **=** 130.6 μM  V_max_ **=** 54.5 nM/s  K_cat_ **=** 0.002729 S^-1^  K_cat_/K_m_ **=** 20.90 S^-1^M^-1^ | IC_50_ = 33.38 ± 2.96 μM Ki = 55.38 ± 23.26 μM | IC_50_ = 9.57 ± 1.42 μM Ki = 16.03 ± 8.58 μM | IC_50_ = 10.01 ± 1.18 μM Ki = 15.52 ± 6.29 μM | IC_50_ = 19.35 ± 2.86 μM Ki = 65.23 ± 29.03 μM |
| H172T + L50F |  | M^pro^ con. = 19.97 μM  K_m_ **=** 143.2 μM  V_max_ **=** 88.88 nM/s  K_cat_ **=** 0.00445 S^-1^  K_cat_/K_m_ **=** 31.08 S^-1^M^-1^ | IC_50_ = 21.09 ± 2.38 μM | IC_50_ = 8.318 ± 0.76 μM | IC_50_ = 7.89 ± 0.63 μM | IC_50_ = 26.01 ± 1.78 μM |
| H172T + T21I |  | M^pro^ con. = 19.97 μM  K_m_ **=** 137.3 μM  V_max_ **=** 123.7 nM/s  K_cat_ **=** 0.006193 S^-1^  K_cat_/K_m_ **=** 45.11 S^-1^M^-1^ | IC_50_ = 29.93 ± 2.70 μM | IC_50_ = 8.809 ± 0.67 μM | IC_50_ = 7.319 ± 0.65 μM | IC_50_ = 19.00 ± 2.53 μM |
| H172T + P132H |  | M^pro^ con. = 19.97 μM  K_m_ **=** 130 μM  V_max_ **=** 68.62 nM/s  K_cat_ **=** 0.003436 S^-1^  K_cat_/K_m_ **=** 26.43 S^-1^M^-1^ | IC_50_ = 17.98 ± 2.27 μM | IC_50_ = 7.656 ± 0.55 μM | IC_50_ = 4.034 ± 0.45 μM | IC_50_ = 8.678 ± 1.1 μM |
| H172E | 16 | M^pro^ con. = 14.793 μM  K_m_ **=** 38.7 μM  V_max_ **=** 14.38 nM/s  K_cat_ **=** 0.0009718 S^-1^  K_cat_/K_m_ **=** 25.11 S^-1^M^-1^ | IC_50_ = 20.66 ± 2.30 μM Ki = 7.508 ± 1.02 μM | IC_50_ = 4.805 ± 0.59 μM Ki = 1.411 ± 0.25 μM | IC_50_ = 4.154 ± 0.56 μM Ki = 1.39 ± 0.25 μM | IC_50_ = 20.9 ± 2.70 μM Ki = 7.519 ± 0.73 μM |
| Q192T | 228 | M^pro^ con. = 1.609 μM  K_m_ **=** 74.02 μM  V_max_ **=** 96.87 nM/s  K_cat_ **=** 0.0602 S^-1^  K_cat_/K_m_ **=** 813.29 S^-1^M^-1^ | IC_50_ = 0.615 ± 0.03 μM Ki = 0.231 ± 0.04 μM | IC_50_ = 0.679 ± 0.02 μM Ki = 0.263 ± 0.04 μM | IC_50_ = 0.459 ± 0.03 μM Ki = 0.179 ± 0.03 μM | IC_50_ = 0.757 ± 0.04 μM Ki = 0.327 ± 0.06 μM |
| Q192K | 80 | M^pro^ con. = 3.362 μM  K_m_ **=** 145.7 μM  V_max_ **=** 150.5 nM/s  K_cat_ **=** 0.04477 S^-1^  K_cat_/K_m_ **=** 307.28 S^-1^M^-1^ | IC_50_ = 1.14 ± 0.07 μM Ki = 0.438 ± 0.11 μM | IC_50_ = 1.279 ± 0.08 μM Ki = 0.505 ± 0.1 μM | IC_50_ = 1.314 μM Ki = 0.360 ± 0.1 μM | IC_50_ = 2.302 ± 0.11 μM Ki = 1.131 ± 0.17 μM |
| Q192Y | 8 | M^pro^ con. = 2.496 μM  K_m_ **=** 133.1 μM  V_max_ **=** 128.8 nM/s  K_cat_ **=** 0.0516 S^-1^  K_cat_/K_m_ **=** 387.68 S^-1^M^-1^ | IC_50_ = 1.065 ± 0.08 μM Ki = 0.382 ± 0.09 μM | IC_50_ = 0.697 ± 0.05 μM Ki = 0.466 ± 0.1 μM | IC_50_ = 0.691 ± 0.06 μM Ki = 0.276 ± 0.07 μM | IC_50_ = 1.531 μM Ki = 0.485 ± 0.12 μM |
| M165Y | 4710 | M^pro^ con. = 5.999 μM  K_m_ **=** 74.71 μM  V_max_ **=** 52.55 nM/s  K_cat_ **=** 0.00876 S^-1^  K_cat_/K_m_ **=** 117.25 S^-1^M^-1^ | IC_50_ = 16.88 ± 0.73 μM Ki = 6.342 ± 0.52 μM | IC_50_ = 1.885 ± 0.06 μM Ki = 0.938 ± 0.15 μM | IC_50_ = 3.111 ± 0.26 μM Ki = 1.432 ± 0.18 μM | IC_50_ = 2.437 ± 0.16 μM Ki = 1.377 ± 0.29 μM |
| M165L | 370 | M^pro^ con. = 211.7 μM  K_m_ **=** 54.61 μM  V_max_ **=** 113.5 nM/s  K_cat_ **=** 0.536 S^-1^  K_cat_/K_m_ **=** 9815.05 S^-1^M^-1^ | IC_50_ = 41.26 ± 2.63 nM Ki = 9.704 ± 2.62 nM | IC_50_ = 114.5 ± 4.92 nM Ki = 33.29 ± 6.64 nM | IC_50_ = 46.74± 2.48 nM Ki = 17.06 ± 3.38 nM | IC_50_ = 130.2 ± 8.16 nM Ki = 57.48 ± 11.73 nM |
| M165K | 10 | M^pro^ con. = 17.751 μM  K_m_ **=** 276.8 μM  V_max_ **=** 105.6 nM/s  K_cat_ **=** 0.005951 S^-1^  K_cat_/K_m_ **=** 21.50 S^-1^M^-1^ | IC_50_ = 14.98 ± 3.2 μM Ki = 12.69 μM | IC_50_ = 5.625 ± 0.46 μM Ki = 5.78 μM | IC_50_ = 8.064 ± 0.85 μM Ki = 8.884 μM | IC_50_ = 16.7 ± 3.43 μM Ki = 9.582 μM |
| E166Q | 4718 | M^pro^ con. = 531.6 nM  K_m_ **=** 377.2 μM  V_max_ **=** 438 nM/s  K_cat_ **=** 0.8239 S^-1^  K_cat_/K_m_ **=** 2184.25 S^-1^M^-1^ | IC_50_ = 0.222 ± 0.03 μM Ki = 76.21 ± 22.8 nM | IC_50_ = 0.369 ± 0.02 μM Ki = 86.6 ± 20.1 nM | IC_50_ = 0.186 ± 0.01 μM Ki = 68.04 ± 18.1 nM | IC_50_ = 0.379 ± 0.03 μM Ki = 102.2 ± 26.6 nM |
| E166Q + L50F |  | M^pro^ con. = 531.6 nM  K_m_ **=** 243.7 μM  V_max_ **=** 398.3 nM/s  K_cat_ **=** 0.7493 S^-1^  K_cat_/K_m_ **=** 3074.68 S^-1^M^-1^ |  |  |  |  |
| E166Q + T21I |  | M^pro^ con. = 531.6 nM  K_m_ **=** 249.1 μM  V_max_ **=** 336.9 nM/s  K_cat_ **=** 0.6338 S^-1^  K_cat_/K_m_ **=** 2544.36 S^-1^M^-1^ |  |  |  |  |
| E166Q + P132H |  | M^pro^ con. = 531.6 nM  K_m_ **=** 324.6 μM  V_max_ **=** 268.3 nM/s  K_cat_ **=** 0.5046 S^-1^  K_cat_/K_m_ **=** 1554.53 S^-1^M^-1^ |  |  |  |  |
| E166H | 331 | M^pro^ con. = 1.156 μM  K_m_ **=** 153.6 μM  V_max_ **=** 195.7 nM/s  K_cat_ **=** 0.1693 S^-1^  K_cat_/K_m_ **=** 1102.21 S^-1^M^-1^ | IC_50_ = 14.65 ± 1.13 μM Ki = 8.306 ± 1.71 μM | IC_50_ = 0.747 ± 0.05 μM Ki = 0.357 ± 0.08 μM | IC_50_ = 0.62 ± 0.05 μM Ki = 0.35 ± 0.06 μM | IC_50_ = 0.7855 ± 0.03 μM Ki = 0.382 ± 0.1 μM |
| E166H + L50F |  | M^pro^ con. = 1.156 μM  K_m_ **=** 138.1 μM  V_max_ **=** 228.1 nM/s  K_cat_ **=** 0.1974 S^-1^  K_cat_/K_m_ **=** 1429.40 S^-1^M^-1^ |  |  |  |  |
| E166H + T21I |  | M^pro^ con. = 1.156 μM  K_m_ **=** 168.3 μM  V_max_ **=** 263.2 nM/s  K_cat_ **=** 0.2278 S^-1^  K_cat_/K_m_ **=** 1353.54 S^-1^M^-1^ |  |  |  |  |
| E166H + P132H |  | M^pro^ con. = 1.156 μM  K_m_ **=** 101.8 μM  V_max_ **=** 89.88 nM/s  K_cat_ **=** 0.07777 S^-1^  K_cat_/K_m_ **=** 763.95 S^-1^M^-1^ |  |  |  |  |
| E166V | 33 | M^pro^ con. = 2.866 μM  K_m_ **=** 277.6 μM  V_max_ **=** 478.5 nM/s  K_cat_ **=** 0.167 S^-1^  K_cat_/K_m_ **=** 601.59 S^-1^M^-1^ | IC_50_ = 47.85 ± 3.82 μM Ki = 6.052 ± 1.65 μM | IC_50_ = 3.103 ± 0.18 μM Ki = 0.56 ± 0.13 μM | IC_50_ = 1.297 ± 0.05 μM Ki = 0.451 ± 0.11 μM | IC_50_ = 4.014 ± 0.20 μM Ki = 1.064 ± 0.29 μM |
| E166V + L50F |  | M^pro^ con. = 2.866 μM  K_m_ **=** 376.3 μM  V_max_ **=** 773.9 nM/s  K_cat_ **=** 0.27 S^-1^  K_cat_/K_m_ **=** 717.51 S^-1^M^-1^ | IC_50_ = 35.32 ± 1.47 μM | IC_50_ = 2.069 ± 0.05 μM | IC_50_ = 1.071 ± 0.04 μM | IC_50_ = 3.653 ± 0.24 μM |
| E166V + T21I |  | M^pro^ con. = 2.866 μM  K_m_ **=** 586 μM  V_max_ **=** 1185 nM/s  K_cat_ **=** 0.4134 S^-1^  K_cat_/K_m_ **=** 705.46 S^-1^M^-1^ | IC_50_ = 72.27 ± 2.70 μM | IC_50_ = 2.451 ± 0.08 μM | IC_50_ = 1.523 ± 0.05 μM | IC_50_ = 4.277 ± 0.30 μM |
| E166V + P132H |  | M^pro^ con. = 2.866 μM  K_m_ **=** 163.5 μM  V_max_ **=** 261.2 nM/s  K_cat_ **=** 0.09113 S^-1^  K_cat_/K_m_ **=** 557.37 S^-1^M^-1^ | IC_50_ = 41.14 ± 2.78 μM | IC_50_ = 3.207 ± 0.19 μM | IC_50_ = 1.176 ± 0.16 μM | IC_50_ = 3.533 ± 0.13 μM |
| E166D | 64 | M^pro^ con. = 4.438 μM  K_m_ **=** 211.6 μM  V_max_ **=** 171.9 nM/s  K_cat_ **=** 0.03873 S^-1^  K_cat_/K_m_ **=** 183.03 S^-1^M^-1^ | IC_50_ = 40.18 ± 3.07 μM Ki = 10.1 ± 3.59 μM | IC_50_ = 1.356 ± 0.09 μM Ki = 0.928 ± 0.19 μM | IC_50_ = 2.704 ± 0.19 μM Ki = 1.886 ± 0.38 μM | IC_50_ = 2.435 ± 0.26 μM Ki = 3.315 ± 0.76 μM |
| E166D + L50F |  | M^pro^ con. = 4.438 μM  K_m_ **=** 156.3 μM  V_max_ **=** 183.7 nM/s  K_cat_ **=** 0.0414 S^-1^  K_cat_/K_m_ **=** 264.88 S^-1^M^-1^ |  |  |  |  |
| E166D + T21I |  | M^pro^ con. = 4.438 μM  K_m_ **=** 253.1 μM  V_max_ **=** 341.1 nM/s  K_cat_ **=** 0.07687 S^-1^  K_cat_/K_m_ **=** 303.71 S^-1^M^-1^ |  |  |  |  |
| E166D + P132H |  | M^pro^ con. = 4.438 μM  K_m_ **=** 143.9 μM  V_max_ **=** 163.9 nM/s  K_cat_ **=** 0.03692 S^-1^  K_cat_/K_m_ **=** 256.57 S^-1^M^-1^ |  |  |  |  |
| E166G | 17 | M^pro^ con. = 485.4 μM  K_m_ **=** 289.1 μM  V_max_ **=** 274.1 nM/s  K_cat_ **=** 0.5648 S^-1^  K_cat_/K_m_ **=** 1953.65 S^-1^M^-1^ | IC_50_ = 0.1823 ± 0.01 μM Ki = 119.4 ± 32.7 nM | IC_50_ = 0.334 ± 0.03 μM Ki = 140.5 ± 27.8 nM | IC_50_ = 0.361 ± 0.02 μM Ki = 179.4 ± 20.7 nM | IC_50_ = 0.257 ± 0.01 μM Ki = 135.5 ± 33.1 nM |
| E166G + L50F |  | M^pro^ con. = 485.4 nM  K_m_ **=** 172 μM  V_max_ **=** 213.1 nM/s  K_cat_ **=** 0.4391 S^-1^  K_cat_/K_m_ **=** 2552.91 S^-1^M^-1^ |  |  |  |  |
| E166G + T21I |  | M^pro^ con. = 485.4 nM  K_m_ **=** 203.2 μM  V_max_ **=** 273.9 nM/s  K_cat_ **=** 0.5642 S^-1^  K_cat_/K_m_ **=** 2776.57 S^-1^M^-1^ |  |  |  |  |
| E166G + P132H |  | M^pro^ con. = 485.4 nM  K_m_ **=** 284.1 μM  V_max_ **=** 165.3 nM/s  K_cat_ **=** 0.3406 S^-1^  K_cat_/K_m_ **=** 1198.87 S^-1^M^-1^ |  |  |  |  |
| E166A | 11 | M^pro^ con. = 665.7 nM  K_m_ **=** 566.6 μM  V_max_ **=** 385 nM/s  K_cat_ **=** 0.5784 S^-1^  K_cat_/K_m_ **=** 1020.83 S^-1^M^-1^ | IC_50_ = 0.376 ± 0.05 μM Ki = 188.3 ± 35.2 nM | IC_50_ = 0.97 ± 0.05 μM Ki = 346.8 ± 56 nM | IC_50_ = 0.2328 ± 0.01 μM Ki = 93.79 ± 20.5 nM | IC_50_ = 0.437 ± 0.03 μM Ki = 211.4 ± 23.6 nM |
| E166A + L50F |  | M^pro^ con. = 665.7 nM  K_m_ **=** 411.1 μM  V_max_ **=** 402.3 nM/s  K_cat_ **=** 0.6044 S^-1^  K_cat_/K_m_ **=** 1470.20 S^-1^M^-1^ |  |  |  |  |
| E166A + T21I |  | M^pro^ con. = 665.7 nM  K_m_ **=** 406.4 μM  V_max_ **=** 368.8 nM/s  K_cat_ **=** 0.554 S^-1^  K_cat_/K_m_ **=** 1363.19 S^-1^M^-1^ |  |  |  |  |
| E166A + P132H |  | M^pro^ con. = 665.7 nM  K_m_ **=** 492.5 μM  V_max_ **=** 219.2 nM/s  K_cat_ **=** 0.3293 S^-1^  K_cat_/K_m_ **=** 668.63 S^-1^M^-1^ |  |  |  |  |
| E166K | 11 | M^pro^ con. =1.942 μM  K_m_ **=** 167.9 μM  V_max_ **=** 86.18 nM/s  K_cat_ **=** 0.04439 S^-1^  K_cat_/K_m_ **=** 264.38 S^-1^M^-1^ | IC_50_ = 3.983 ± 0.38 μM Ki = 2.494 ± 0.6 μM | IC_50_ = 8.831 ± 0.7 μM Ki = 6.481 ± 1.27 μM | IC_50_ = 1.469 μM Ki = 0.785 ± 0.22 μM | IC_50_ = 2.521 ± 0.16 μM Ki = 1.572 ± 0.46 μM |
| E166del | 44 | M^pro^ con. = 19.97 μM  K_m_ **=** 81.69 μM  V_max_ **=** 2.549 nM/s  K_cat_ **=** 0.0001276 S^-1^  K_cat_/K_m_ **=** 1.56 S^-1^M^-1^ | IC_50_ > 200μM | | | |
| SARS-1 |  |  | IC_50_ = 174.3 ± 11.53 nM | IC_50_ = 134.6 ± 5.79 nM | IC_50_ = 73.01 ± 2.46 nM | IC_50_ = 243.9 ± 40.2 nM |
| MERS |  |  | IC_50_ = 951.7 ± 128.4 nM | IC_50_ = 1.756 ± 0.14 μM | IC_50_ = 945.3 ± 66.3 nM | IC_50_ = 2.453 ± 0.29 μM |
| HKU1 |  |  | IC_50_ = 76.92 ± 7.52 nM | IC_50_ = 72.79 ± 6.54 nM | IC_50_ = 52.34 ± 2.84 nM | IC_50_ = 73.26 ± 7.73 nM |
| OC43 |  |  | IC_50_ = 92.1 ± 5.36 nM | IC_50_ = 158.2 ± 12.4 nM | IC_50_ = 53.18 ± 2.78 nM | IC_50_ = 136.7 ± 15.54 nM |
| 229E |  |  | IC_50_ = 422.7 ± 37.7 nM | IC_50_ = 6.11 ± 0.99 μM | IC_50_ = 100.5 ± 9.47 nM | IC_50_ = 238.9 ± 15.2 nM |
| NL63 |  |  | IC_50_ = 1.304 ± 0.1 μM | IC_50_ = 82.43 ± 12.43 μM | IC_50_ = 271.7 ± 26.48 nM | IC_50_ = 438.4 ± 49.87 nM |
